# Cooperative Carbon Dioxide Capture in Diamine-Appended Magnesium–Olsalazine Frameworks

**DOI:** 10.1021/jacs.3c03870

**Published:** 2023-07-26

**Authors:** Ziting Zhu, Surya T. Parker, Alexander C. Forse, Jung-Hoon Lee, Rebecca L. Siegelman, Phillip J. Milner, Hsinhan Tsai, Mengshan Ye, Shuoyan Xiong, Maria V. Paley, Adam A. Uliana, Julia Oktawiec, Bhavish Dinakar, Stephanie A. Didas, Katie R. Meihaus, Jeffrey A. Reimer, Jeffrey B. Neaton, Jeffrey R. Long

**Affiliations:** † Department of Materials Science and Engineering, University of California, Berkeley, California94720, United States; ‡ Department of Chemical and Biomolecular Engineering, University of California, Berkeley, California94720, United States; § Department of Chemistry, University of California, Berkeley, California94720, United States; ∥ Department of Physics, University of California, Berkeley, California94720, United States; ⊥ Molecular Foundry, Lawrence Berkeley National Laboratory, Berkeley, California 94720, United States; # Materials Sciences Division, Lawrence Berkeley National Laboratory, Berkeley, California 94720, United States

## Abstract

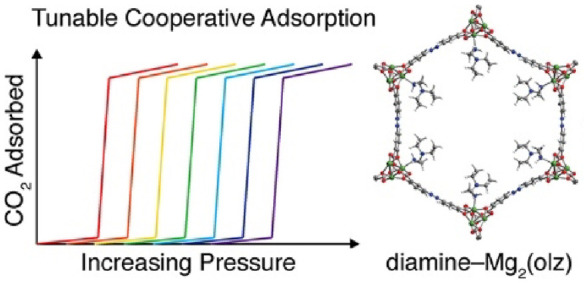

Diamine-appended Mg_2_(dobpdc) (dobpdc^4–^ = 4,4′-dioxidobiphenyl-3,3′-dicarboxylate) metal–organic frameworks have emerged as promising candidates for carbon capture owing to their exceptional CO_2_ selectivities, high separation capacities, and step-shaped adsorption profiles, which arise from a unique cooperative adsorption mechanism resulting in the formation of ammonium carbamate chains. Materials appended with *primary*,*secondary*-diamines featuring bulky substituents, in particular, exhibit excellent stabilities and CO_2_ adsorption properties. However, these frameworks display double-step adsorption behavior arising from steric repulsion between ammonium carbamates, which ultimately results in increased regeneration energies. Herein, we report frameworks of the type diamine–Mg_2_(olz) (olz^4–^ = (*E*)-5,5′-(diazene-1,2-diyl)bis(2-oxidobenzoate)) that feature diverse diamines with bulky substituents and display desirable single-step CO_2_ adsorption across a wide range of pressures and temperatures. Analysis of CO_2_ adsorption data reveals that the basicity of the pore-dwelling amine—in addition to its steric bulk—is an important factor influencing adsorption step pressure; furthermore, the amine steric bulk is found to be inversely correlated with the degree of cooperativity in CO_2_ uptake. One material, ee-2–Mg_2_(olz) (ee-2 = *N*,*N*-diethylethylenediamine), adsorbs >90% of the CO_2_ from a simulated coal flue stream and exhibits exceptional thermal and oxidative stability over the course of extensive adsorption/desorption cycling, placing it among top-performing adsorbents to date for CO_2_ capture from a coal flue gas. Spectroscopic characterization and van der Waals-corrected density functional theory calculations indicate that diamine–Mg_2_(olz) materials capture CO_2_ via the formation of ammonium carbamate chains. These results point more broadly to the opportunity for fundamentally advancing materials in this class through judicious design.

## Introduction

Anthropogenic carbon dioxide emissions are largely responsible for the deleterious global warming measured to date above preindustrial levels, and this warming is rapidly approaching the 1.5 °C increase that is predicted to cause a global climate crisis.^1^ Emissions from fossil fuel combustion and industrial processes accounted for nearly 90% of all greenhouse gases emitted in 2021,^2^ and coal-fired power stations alone are responsible for 30% of all the CO_2_ emissions.^3^ Although a shift toward greater usage of renewable and carbon-free energy sources, such as solar and wind, is underway, fossil fuels are projected to continue as a dominant global energy source through 2050.^4^ As a result, carbon capture and sequestration (CCS) is widely acknowledged to be a critical strategy for mitigating CO_2_ emissions in the near term, while the use of fossil fuels continues.^5,6^ Furthermore, considering industries such as cement and steel manufacturing, where decarbonization cannot be achieved through a transition away from fossil fuels, CCS will be particularly critical to reduce CO_2_ emissions in the long term. Approximately 60–70% of the total cost of CCS is associated with CO_2_ capture,^7^ resulting in an urgent need for novel CO_2_ capture materials with high CO_2_ selectivities, large separation working capacities, and low regeneration energies. Target gas streams for carbon capture also contain different concentrations of CO_2_ depending on the point source. For example, a typical flue gas composition from a coal-fired power station contains 15–16% CO_2_, while natural gas combined cycle power plants produce flue gas with a lower CO_2_ concentration of ∼4%.^8,9^ Beyond the power sector, upgrading crude biogas to biomethane requires capture of CO_2_ at higher concentrations (30–60%) from CH_4_, and the cement industry produces waste gas streams with ∼30% CO_2_.^10,11^

Aqueous amine solutions represent the current state-of-the-art technology for postcombustion CO_2_ capture, and these operate by selectively absorbing CO_2_ to form ammonium carbamate and bicarbonate species.^12^ However, such solutions exhibit low working capacities and high regeneration energies, are susceptible to oxidative and thermal degradation,^12−15^ and can cause significant carbon steel corrosion.^16^ Porous materials including activated carbons and zeolites have been investigated as alternatives for CO_2_ separations, owing to their large internal surface areas, high adsorption capacities, robust structures, and relatively low regeneration energies.^17−23^ However, most porous materials are not suitable for industrial separations due to the fact that water vapor in flue gas outcompetes CO_2_ at the material binding sites.^17−23^ To address this issue, amine-functionalized silicas^24−28^ and metal–organic frameworks (MOFs)^29,30^ have been developed as promising alternatives. Analogous to the selective chemisorption of CO_2_ in aqueous amine solutions, in these materials, the appended amines selectively react with CO_2_ to form ammonium carbamate and/or bicarbonate species, even in the presence of water vapor.^24,29^

**Figure 1:**
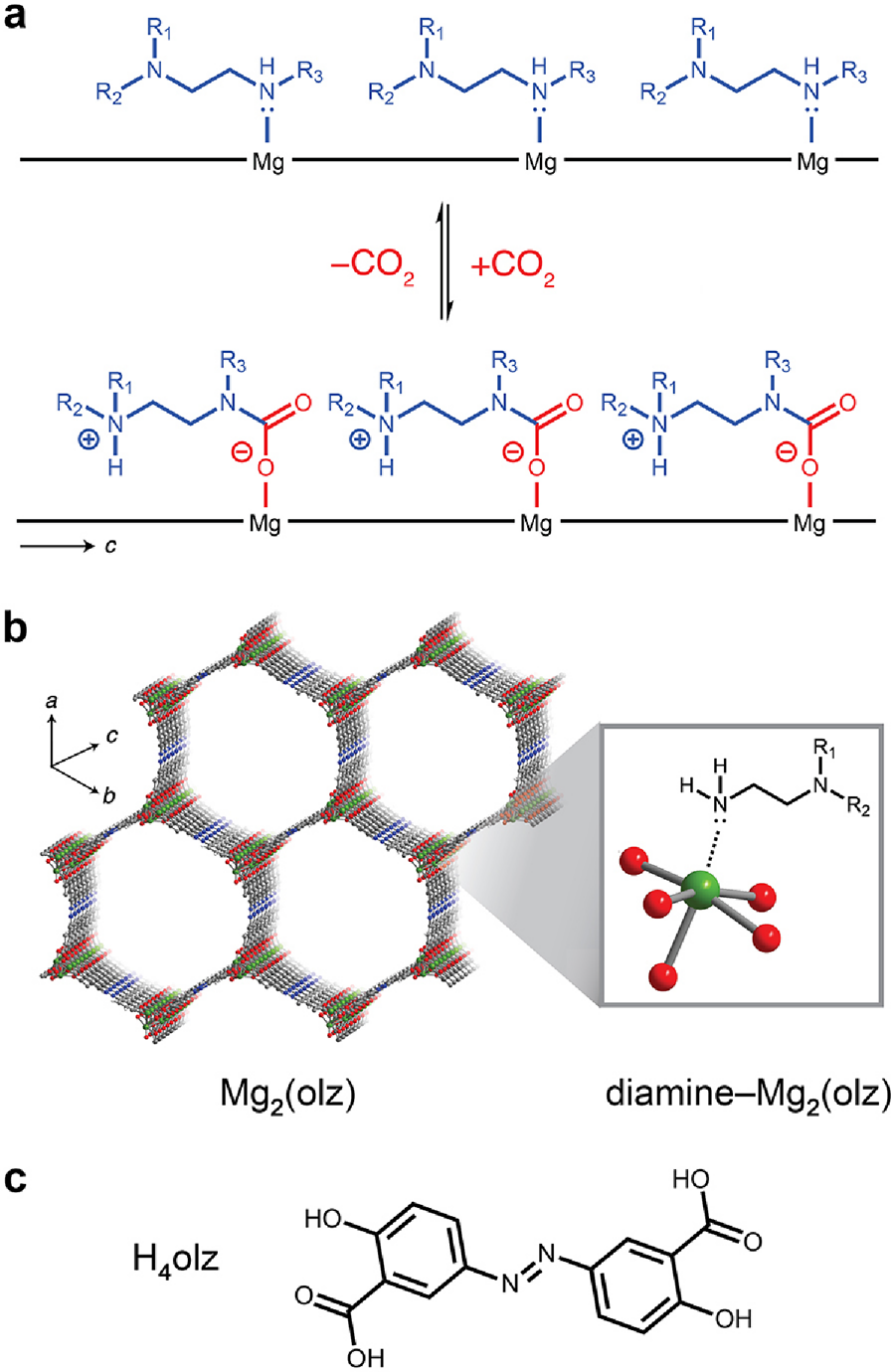
(a) Depiction of cooperative CO_2_ insertion into diamine–Mg_2_(dobpdc) to form chains of ammonium carbamate. (b) Structure of activated Mg_2_(olz), which was postsynthetically functionalized with diamines to generate diamine–Mg_2_(olz). Green, red, blue, gray, and white depict the Mg, N, C, and H atoms, respectively. (c) Structure of the H_4_olz linker.

Among such materials, alkyldiamine-functionalized frameworks of the type diamine–Mg_2_(dobpdc) (dobpdc^4–^ = 4,4′-dioxidobiphenyl-3,3′-dicarboxylate)^31−42^ exhibit exceptional selectivities and capacities for CO_2_, arising from a unique cooperative adsorption mechanism wherein CO_2_ molecules insert into the framework metal–amine bonds to form ammonium carbamate chains that propagate down the one-dimensional channels (Figure 1a).^32^ This mechanism is associated with step-shaped adsorption profiles in which CO_2_ uptake to near saturation occurs within a narrow temperature or pressure range.^32,33^ An analogous mechanism has also been shown to be operative in robust tetraamine–Mg_2_(dobpdc) materials that are remarkably stable even to steam regeneration.^43^ To date, the most prevalent strategy for tuning the threshold for CO_2_ adsorption in diamine–Mg_2_(dobpdc) materials has been to vary the diamine, and the CO_2_ adsorption properties of Mg_2_(dobpdc) appended with *primary*,*secondary* (1°,2°)-, *primary*,*tertiary* (1°,3°)-, and *secondary*,*secondary* (2°,2°)-diamines have been studied in detail. Materials featuring 1°,2°-diamines have emerged as frontrunners for applications in CO_2_ capture from coal (and natural gas) flue gas, given their thermal stabilities and low CO_2_ adsorption pressures under isothermal conditions.^31−38^

For 1°,2°-diamine-appended Mg_2_(dobpdc) materials, increasing the steric bulk of the pore-dwelling secondary amine has been shown to further enhance stability to adsorption/desorption cycling.^38^ However, these frameworks exhibit CO_2_ adsorption profiles with two steps rather than one, which has been attributed to steric conflict between adjacent ammonium carbamate chains in the *ab* crystal plane due to the asymmetric pore structure. This phenomenon limits the overall CO_2_ capacity under a given set of conditions and leads to higher regeneration energies.^38^ Changing the base framework from Mg_2_(dobpdc) to Mg_2_(pc-dobpdc) (pc-dobpdc^4–^ = 3,3′-dioxidobiphenyl-4,4′-dicarboxylate, pc = para-carboxylate) or Mg_2_(dotpdc) (dotpdc^4–^ = 4,4″-dioxido-[1,1′:4′,1′′-terphenyl]-3,3′′-dicarboxylate) alleviates the steric conflict, giving rise to materials that exhibit single-step CO_2_ adsorption profiles.^38^ However, as a result of the larger size of the dotpdc^4–^ linker, diamine–Mg_2_(dotpdc) materials display gravimetric capacities lower than diamine–Mg_2_(dobpdc) materials. Furthermore, an expensive palladium catalyst is required for the synthesis of H_4_pc-dobpdc and H_4_dotpdc linkers, rendering the corresponding amine-appended frameworks impractical for large-scale use.

Seeking to overcome the aforementioned limitations of the bulky 1°,2°-diamine-appended Mg_2_(dobpdc) materials, we were interested in identifying a MOF that, when appended with diamines, would exhibit robust, tunable single-step CO_2_ adsorption unperturbed by changes to the steric bulk of the pore-dwelling amine. We chose to investigate Mg_2_(olz) (H_4_olz = olsalazine; Figure 1b,c), which is an expanded pore analogue of Mg_2_(dobpdc) previously developed as a biocompatible platform for drug delivery.^44^ Of note, the olz^4–^ linker is slightly longer than dobpdc^4–^ but shorter than dotpdc^4–^, and its synthesis does not require the use of an expensive precious metal catalyst. Herein, we report a family of diamine–Mg_2_(olz) frameworks that exhibit cooperative CO_2_ uptake for a range of 1°,1°-, 1°,2°-, and 1°,3°-diamines, as well as higher volumetric capacities than related materials prepared with Mg_2_(dotpdc).^38^ Importantly, all the diamine–Mg_2_(olz) materials exhibit single-stepped adsorption behavior, including those appended with bulky 1°,2°-diamines, in contrast to previously reported diamine–Mg_2_(dobpdc) analogues.^35^ Further, by tuning the diamine steric bulk and the basicity of the pore-dwelling amine, it is possible to tune the CO_2_ step pressure of diamine–Mg_2_(olz) over three orders of magnitude, and as such these materials exhibit unparalleled versatility for CO_2_ capture from numerous target emission streams. The variant ee-2–Mg_2_(olz) (ee-2 = *N*,*N*-diethylethylenediamine) in particular exhibits exceptional CO_2_ adsorption properties relevant to carbon capture from coal flue gas.

## Results and Discussion

### Framework Synthesis and CO_2_ Adsorption Properties

The framework Mg_2_(olz) was prepared via a modified version of the previously reported synthesis (see the Experimental Section and Figures S1–S4).^44^ Diamine grafting was accomplished by soaking methanol-solvated Mg_2_(olz) with the diamine of choice in toluene for several hours. We selected one 1°,1°-diamine, 1,2-diamino-2-methylpropane (dmen),^34,36^ and eight 1°,2°- and 1°,3°-diamines featuring variously substituted secondary or tertiary amines (Table 1) to rigorously evaluate the performance of diamine–Mg_2_(olz) frameworks for CO_2_ capture and the impact of diamine structure on CO_2_ adsorption performance. We adopt a previously described shorthand^33^ for each diamine that first specifies the alkyl substituent(s) on the pore-dwelling amine, the number of carbons in the alkyl bridge, and the substituent on the metal-bound amine (as relevant). For example, the shorthand for the 1°,2°-diamine *N*,*N*-dimethylethylenediamine is mm-2. Previous analysis of the structures of several diamine–Zn_2_(dobpdc) variants using single-crystal X-ray diffraction revealed that 1°,2°- and 1°,3°-diamines typically bind to the framework metal sites via the primary amine,^33^ and this is presumed to be the case for diamine–Mg_2_(olz) as well. Langmuir surface areas were calculated from 77 K N_2_ adsorption data, and diamine loadings were confirmed by ^1^H NMR spectroscopy analysis of digested framework samples (Tables S1 and S2, respectively). The materials form as microcrystalline solids (Figure S4 and S6) with decomposition temperatures exceeding 200 °C (Figure S7).

**Table 1:**
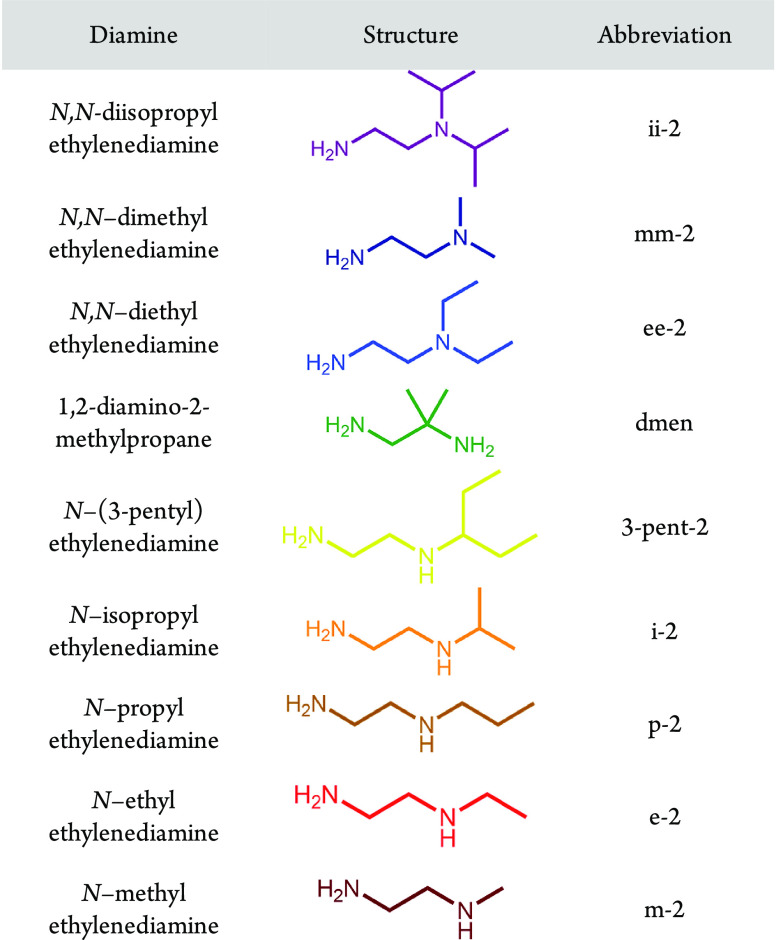
Structures and Shorthand for Diamines Used in This Work

**Figure 2:**
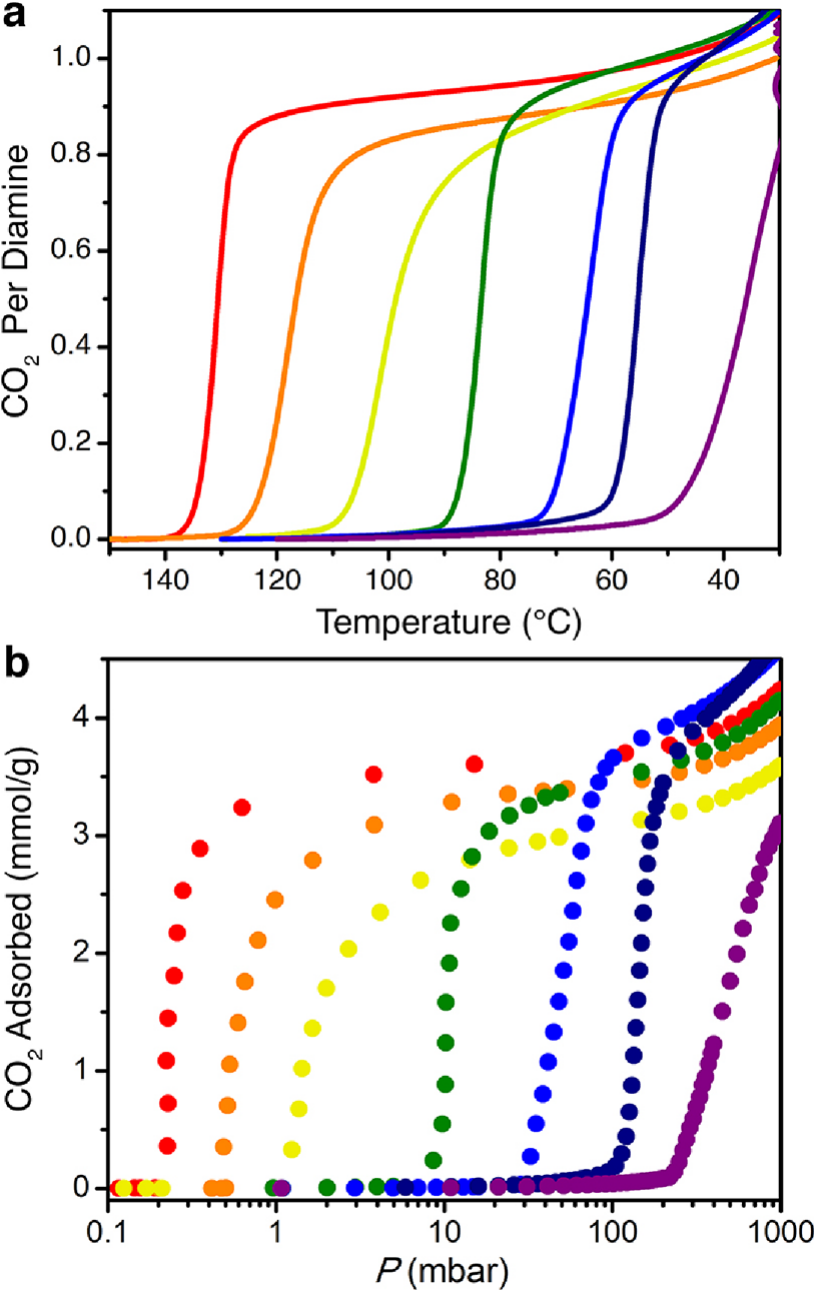
(a) Adsorption isobars (from left to right) obtained for e-2–, i-2–, 3-pent-2–, dmen–, ee-2–, mm-2–, and ii-2–Mg_2_(olz) under pure CO_2_, as measured by thermogravimetric analysis. (b) Pure CO_2_ adsorption isotherms (from left to right) obtained at 40 °C for e-2–, i-2–, 3-pent-2–, dmen–, ee-2–, mm-2–, and ii-2–Mg_2_(olz). The data for m-2–Mg_2_(olz) and p-2–Mg_2_(olz) nearly overlay those collected for e-2–Mg_2_(olz) and are omitted here for simplicity. See Figure 3a for the corresponding isotherms at 85 °C for all three 1°,2°-diamines bearing linear alkyl substituents.

Thermogravimetric CO_2_ adsorption isobars and CO_2_ adsorption isotherms were collected for the diamine–Mg_2_(olz) compounds under an atmosphere of pure CO_2_. All variants exhibit step-shaped CO_2_ uptake under isobaric and isothermal conditions (Figure 2 and Figure S8), consistent with cooperative adsorption and ammonium carbamate chain formation.^31−38^ The frameworks also retain their crystallinity and underlying structure upon pure CO_2_ adsorption, as determined from *in situ* powder X-ray diffraction analysis (Figure S6). For each material, the CO_2_ uptake under isobaric conditions begins to plateau near the theoretical capacity of one CO_2_ molecule per diamine (Figure 2a), followed by a more gradual CO_2_ uptake due to physisorption. Importantly, all of the diamine–Mg_2_(olz) frameworks exhibit single-step adsorption profiles, regardless of the size of the alkyl substituent on the pore-dwelling amine (Figures S8–S10). In contrast, prior work has shown that p-2-, i-2-, 3-pent-2-, and dmen-appended Mg_2_(dobpdc) exhibit double-stepped adsorption behavior,^33,35,36^ and CO_2_ adsorption to full capacity (one CO_2_ per diamine) in the case of dmen–Mg_2_(dobpdc) is also kinetically limited,^36^ which is not the case for dmen–Mg_2_(olz). We attribute the single-step behavior for these diamine–Mg_2_(olz) materials to the absence of steric hindrance between neighboring ammonium carbamate chains formed upon CO_2_ uptake.

The CO_2_ adsorption step temperatures for diamine–Mg_2_(olz) range from 35 to 135 °C (Figure 2a). Given the different adsorption profiles for some of these materials, in contrast with their diamine–Mg_2_(dobpdc) counterparts exhibiting two-stepped adsorption, it is challenging to make any direct comparisons. However, the relative step positions for diamine–Mg_2_(olz) trend with the first adsorption step positions for the corresponding diamine–Mg_2_(dobpdc) materials. In the case of p-2– and i-2–Mg_2_(olz), adsorption occurs at a slightly higher temperature than initial adsorption in p-2– and i-2–Mg_2_(dobpdc),^38^ while the opposite is true for 3-pent-2–^38^ and dmen–Mg_2_(olz).^36^ Relative to diamine–Mg_2_(dobpdc) variants exhibiting single-stepped adsorption (those featuring m-2, e-2, mm-2, ii-2, and ee-2), the corresponding diamine–Mg_2_(olz) variants again generally exhibit consistent trends—1°,2°-diamine-appended variants adsorb CO_2_ with step temperatures above 120 °C, while 1°,3°-diamine-appended variants adsorb CO_2_ with step positions below 70 °C.^33^ Finally, for diamine–Mg_2_(olz), the step pressure at 40 °C can be tuned over three orders of magnitude, from 0.2 to 200 mbar (Figure 2b). Significantly, these step positions span the range of the CO_2_ partial pressures for most target flue gases for carbon capture. Further, ee-2–, dmen–, 3-pent-2–, i-2–, and e-2–Mg_2_(olz) exhibit step pressures below 150 mbar, and as such, they are all potential candidates for CO_2_ capture from a coal flue gas.

**Figure 3:**
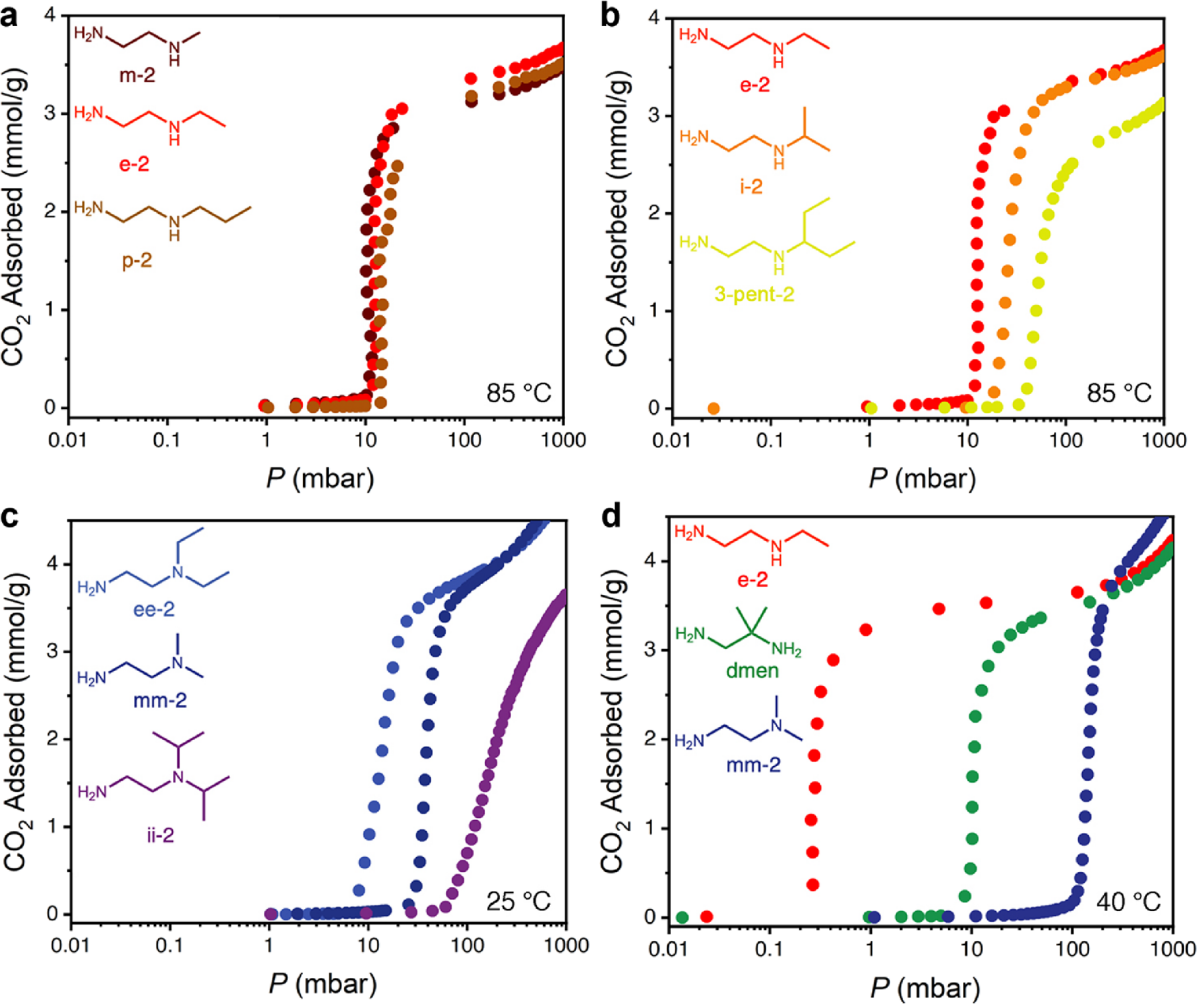
Comparisons of adsorption isotherms under pure CO_2_ for different series of diamine–Mg_2_(olz) variants illustrating the effect of the diamine structure on the adsorption step pressure. (a) 1°,2°-Diamines bearing linear alkyl substituents, (b) 1°,2°-diamines with increasingly branched substituents, (c) 1°,3°-diamines with different degrees of substituent branching, and (d) diamines with two-carbon alkyl substitutions. Note the distinct isotherm temperatures for each series: data were collected at 85 °C for panels (a) and (b), 25 °C for panel (c), and 40 °C for panel (d).

In the case of diamine–Mg_2_(olz) variants featuring 1°,2°-diamines, increasing the secondary amine alkyl chain length from one carbon (m-2) to three carbons (p-2) has little effect on the CO_2_ step pressure (defined here as the inflection point of the adsorption step), which changes from 10.2 to 13.9 mbar at 85 °C (Figure 3a). In contrast, branched alkyl groups have a more substantial effect on CO_2_ adsorption, as illustrated by the increase in step pressure from 12.4 mbar for e-2–Mg_2_(olz) to 52.6 mbar for 3-pent-2–Mg_2_(olz) (Figure 3b). Along with this step pressure increase, the slope of the step decreases slightly as the size of the alkyl group increases, which is indicative of a reduction in the degree of cooperativity in CO_2_ uptake (discussed further below).^45^ Overall, the increasing step pressure in the adsorption isotherms (and decreasing step temperature in adsorption isobars; see Figure S8) with increasing substituent size is consistent with weaker ammonium carbamate ion pairing as the secondary amines become more sterically encumbered.^37^

Interestingly, for 1°,3°-diamine–Mg_2_(olz) materials, there is not a consistent correlation between the CO_2_ step pressure and the steric bulk of the tertiary amine substituent (Figure 3c). Indeed, although the step pressure of ii-2–Mg_2_(olz) is higher than that of mm-2–Mg_2_(olz) (174 versus 38.7 mbar) at 25 °C, as might be expected based on sterics, the step pressure for ee-2–Mg_2_(olz) is lower than that of mm-2–Mg_2_(olz) (13.8 versus 38.7 mbar), even though ee-2 features bulkier alkyl groups. It is also interesting to compare the isothermal (40 °C) step pressures for e-2–, dmen–, and mm-2–Mg_2_(olz), which feature diamines with the same overall number of carbon atoms but differing degrees of steric bulk. Based on steric hindrance alone, we might expect that dmen–Mg_2_(olz) would exhibit the lowest step pressure; however, its step pressure (10.2 mbar) is intermediate between those of e-2– and mm-2–Mg_2_(olz) (0.3 and 141 mbar, respectively; Figure 3d). Step temperatures determined from isobaric measurements followed the same trends (Figure S8).

It is clear that the structures and steric bulk of the pore-dwelling amine impact the CO_2_ adsorption properties of diamine–Mg_2_(olz), although these aspects alone do not fully explain the trends observed. Another important factor is the basicity of the pore-dwelling amine, which abstracts a proton from the metal-bound amine concomitant with CO_2_ insertion.^32^ We therefore sought to investigate whether there is any correlation between the basicity of the free amine and the observed step pressure or temperature for CO_2_ adsorption. To this end, we generated a series of monoamines to represent the pore-dwelling primary, secondary, or tertiary amine (Table S3) by replacing the metal-bound primary amine with a proton (the least sterically hindered primary amine in the case of dmen). We found experimental p*K*_a_ values only for a select few of the corresponding ammonium cations,^46^ and therefore calculated p*K*_a_ values were also generated for all the representative ammonium cations using SciFinder.^47^ The calculated p*K*_a_ values were found to be very similar to the available experimental values (Table S3). Below, we compare experimental data when they are available for all amines under consideration, and otherwise calculated values are discussed.

For the secondary amines with straight chain alkyl groups, increasing the length of the linear alkyl group from one to three carbons does not significantly change the basicity of the amine (calculated p*K*_a_ values for the representative ammonium cations range from 10.8(1) to 10.8(2); Table S3). As such, for m-2–, e-2–, and p-2–Mg_2_(olz), steric hindrance appears to be the primary factor influencing the (small) differences in their adsorption step positions. Interestingly, the tertiary amines in ee-2 and ii-2 are both expected to be more basic than the tertiary amine in mm-2 (the calculated p*K*_a_ values for the representative ammonium cations are 10.6(3), 11.0(3), and 9.8(3), respectively). The greater basicity of the tertiary amine in ee-2 could explain why ee-2–Mg_2_(olz) exhibits a lower step pressure (more favorable CO_2_ uptake) than mm-2–Mg_2_(olz), despite having a more sterically encumbered free amine. However, the step pressure of ii-2–Mg_2_(olz) is the highest of all three materials, even though its tertiary amine is predicted to be the most basic. This result suggests that significant steric bulk can counteract basicity as a driving force for CO_2_ binding. Finally, for e-2–, dmen–, and mm-2–Mg_2_(olz), the CO_2_ step pressure seems to directly correlate with the basicity of the representative pore-dwelling amine (the experimental p*K*_a_ values for the representative ammonium cations are 10.98, 10.45, and 9.99, respectively), although sterics cannot be ruled out as a mitigating factor.

In all, these results suggest that when designing diamine-appended frameworks for CO_2_ capture from a specific target stream, it would be valuable to consider both the steric hindrance from branching alkyl groups on the pore-dwelling amine and the basicity of the pore-dwelling amine. Our results indicate that materials with more basic pore-dwelling amines are likely to perform better for the capture of CO_2_ at high temperatures (isobaric conditions) or low pressures (isothermal conditions). However, branching alkyl groups can counteract the effect of basicity by destabilizing the ammonium carbamate chain phase, and as such, the size of the alkyl substituent should also be taken into account.

### Cooperativity of CO_2_ Adsorption

As noted above, we observed that increasing the steric bulk of the secondary or tertiary amine in 1°,2°- and 1°,3°-amine-appended Mg_2_(olz) leads to a decrease in the sharpness of the CO_2_ adsorption step (Figure 3b,d), which is indicative of a reduction in the degree of cooperativity in CO_2_ uptake. To quantify this change and elucidate any trends relating the diamine structure to degree of cooperativity, we analyzed diamine–Mg_2_(olz) isotherm data collected at various temperatures using the Hill equation (see Section 2 of the Supporting Information for details).^45^ The Hill coefficient obtained for each material can be viewed as an approximation of the number of CO_2_ molecules involved in cooperative ammonium carbamate chain formation. While this analysis does not allow for an absolute description of the cooperativity of CO_2_ binding in a given framework, it is useful to establish overall trends, as previously demonstrated for *N*,*N*′-dimethylethylenediamine-appended M_2_(dobpdc) (M = Mg, Mn, Fe, Co, Ni, and Zn).^32^

For each of the diamine–Mg_2_(olz) materials, neither the Hill coefficient nor the slope of the CO_2_ isotherm was found to change significantly with an increase in temperature (Table S4). Thus, for a given framework, the degree of cooperativity in CO_2_ uptake is relatively insensitive to the temperature over the examined range. However, a comparison of diamines within the same family (1°,2°- or 1°,3°-diamines) reveals that the sharpness of the step is dependent on the diamine structure. For m-2–Mg_2_(olz) and e-2–Mg_2_(olz), the estimated Hill coefficients (*n*) are both 11(4), whereas increasing the alkyl chain length to three carbons in p-2–Mg_2_(olz) results in a decrease of *n* to 6(2). For frameworks appended with branching 1°,2°- or 1°,3°-diamines, *n* decreases with increasing steric bulk from 11(4) to 7(1) to 6(2) for e-2–Mg_2_(olz), i-2–Mg_2_(olz), and 3-pent-2–Mg_2_(olz), respectively, and from 9(1) to 4.9(6) to 3.0(1) for mm-2–Mg_2_(olz), ee-2–Mg_2_(olz), and ii-2–Mg_2_(olz), respectively. Interestingly, even though the CO_2_ adsorption step temperature of ee-2–Mg_2_(olz) is higher than that of mm-2–Mg_2_(olz), and therefore the initial CO_2_ uptake is more thermodynamically favorable in ee-2–Mg_2_(olz), CO_2_ adsorption in mm-2–Mg_2_(olz) is more cooperative. Altogether, these results suggest that the diamine structure is a dominant factor influencing the degree of cooperativity in CO_2_ uptake in these materials.

**Figure 4:**
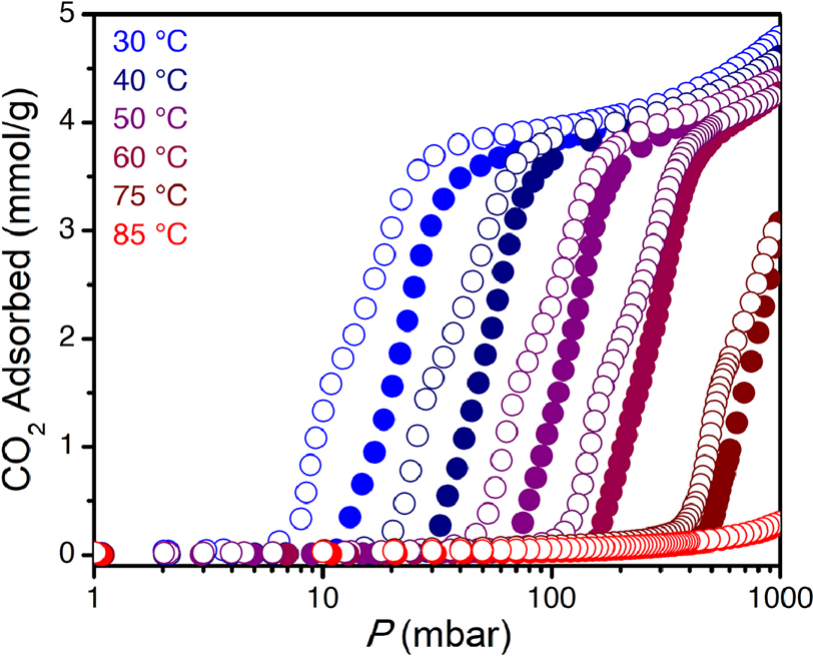
Carbon dioxide adsorption isotherms for ee-2–Mg_2_(olz) collected at the indicated temperatures with pressure plotted on a logarithmic scale. The filled and empty circles represent the adsorption and desorption data, respectively.

### Evaluation of Diamine–Mg_2_(olz) Coal Flue Gas Capture

Based on adsorption isotherm data, ee-2–, dmen–, 3-pent-2–, i-2–, and e-2–Mg_2_(olz) are all candidates for the removal of CO_2_ from a coal flue gas, owing to their CO_2_ step pressures below 150 mbar at 40 °C. Of these materials, ee-2–Mg_2_(olz) exhibits the lowest desorption temperature of 85 °C, which prompted us to examine its temperature-dependent CO_2_ adsorption properties in more detail. Accordingly, CO_2_ adsorption and desorption isotherms were collected at temperatures ranging from 30 to 85 °C. The material exhibits step-shaped adsorption at temperatures ≤75 °C, and the step pressure increases with increasing temperature (Figure 4). Consistent with the isobaric data (Figure S10), the CO_2_ isotherm for ee-2–Mg_2_(olz) at 85 °C is nearly flat up to a pressure of 1 bar (Figure S12). Based on single-component isotherm data collected for ee-2–Mg_2_(olz), adsorption of CO_2_ at 150 mbar and 40 °C and desorption under 1 bar of CO_2_ at 85 °C would yield a high working capacity of 3.53 mmol/g or 15.5 wt % (Figure 4). Using the crystallographic density of activated ee-2–Mg_2_(olz) (0.744 g/cm^3^) (Table S8), this corresponds to an approximate volumetric working capacity of 2.63 mmol/cm^3^ (59 v/v).

We calculated the differential enthalpy of CO_2_ adsorption in ee-2–Mg_2_(olz) as a function of loading from linear interpolation of the CO_2_ adsorption isotherms using the Clausius–Clapeyron relationship (see the Experimental Section).^48^ At a CO_2_ loading of 2 mmol/g (i.e., the midpoint of the adsorption step), the differential enthalpy of CO_2_ adsorption (Δ*h*_ads_) is −69.9 ± 0.8 kJ/mol. Using the low reversible heat capacity (2.02 J/g·°C) of ee-2–Mg_2_(olz) measured by differential scanning calorimetry (DSC) (Figure S20), we calculated an approximate regeneration energy of 2.15 MJ/kg of CO_2_ (see Section 3.3 of the Supporting Information for details). Significantly, this value is 40% lower than that for monoethanolamine (3.6 MJ/kg CO_2_)^49^ and 15% less than the regeneration energy associated with the framework dmpn–Mg_2_(dobpdc) (2.53 MJ/kg CO_2_; dmpn = 2,2-dimethyl-1,3-diaminopropane), the leading diamine-appended Mg_2_(dobpdc) framework for CO_2_ capture from a coal flue gas.^34^ Additionally, monoethanolamine and dmpn–Mg_2_(dobpdc) must be heated to significantly higher temperatures (100–130 °C) than ee-2–Mg_2_(olz) to fully desorb CO_2_ and would therefore require the use of high-value steam for regeneration.

**Figure 5:**
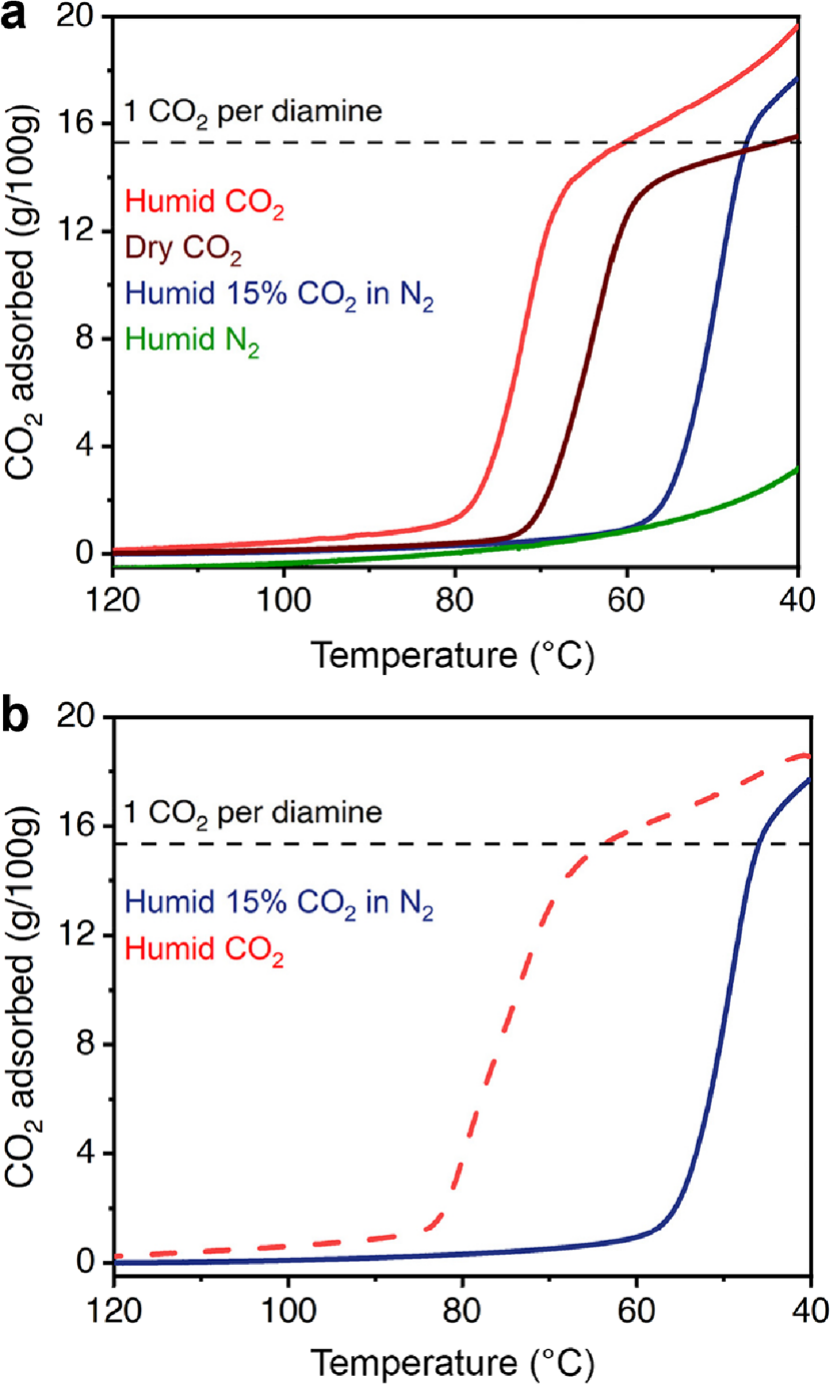
(a) Comparison of humid CO_2_ (∼1.5% H_2_O), dry CO_2_, humid 15% CO_2_ in N_2_ (∼1.5% H_2_O), and humid N_2_ (∼1.5% H_2_O) adsorption isobars for ee-2–Mg_2_(olz) at atmospheric pressure. (b) Humid 15% CO_2_ in N_2_ (∼1.5% H_2_O) adsorption isobar (cooling, solid blue line) and humid CO_2_ (∼1.5% H_2_O) desorption isobar (heating, dashed red line) for ee-2–Mg_2_(olz) under atmospheric pressure. A ramp rate of 1 °C/min was used for all of the isobaric experiments.

### Adsorption Performance of ee-2–Mg_2_(olz) under Simulated Humid Flue Gas

We next sought to evaluate the CO_2_ adsorption performance of ee-2–Mg_2_(olz) under more realistic multicomponent conditions. To this end, we carried out thermogravimetric analysis (TGA) experiments wherein a sample of the framework was exposed to a humidified (∼1.5% H_2_O) stream containing 15% CO_2_ in N_2_ (see the Experimental Section for details). Because this experiment enables determination of only the total quantity of adsorbed gases, we also carried out separate experiments using humidified CO_2_ or humidified N_2_ (∼1.5% H_2_O in each case) to enable a qualitative assessment of the adsorption performance under a simulated humid flue gas (Figure 5a). Isobaric adsorption data obtained for a sample of ee-2–Mg_2_(olz) dosed with humid N_2_ (Figure 5a, green curve) revealed gradual (i.e., nonstepped) gas uptake to only 3.03 g per 100 g of adsorbent at 40 °C. Given that ee-2–Mg_2_(olz) adsorbs negligible N_2_ under dry isothermal and isobaric conditions (Figures S16 and S17), the adsorbed mass under humid N_2_ can be attributed to uptake of water only (approximately 0.47 molecules of water adsorbed per diamine).

When dosed with dry, pure CO_2_ under isobaric conditions, ee-2–Mg_2_(olz) exhibits step-shaped gas uptake with a step temperature of ∼65 °C and adsorbs 15.5 g/100 g (3.53 mmol/g) at 40 °C (Figure 5a, burgundy curve), consistent with the theoretical loading for adsorption of 1 CO_2_ per diamine (15.2 g/100 g or 3.45 mmol/g). When exposed to a stream of humid (∼1.5% H_2_O) CO_2_, ee-2–Mg_2_(olz) also exhibits step-shaped gas uptake, albeit with a higher onset temperature of 80 °C. This result suggests that water promotes CO_2_ uptake in ee-2–Mg_2_(olz), a phenomenon observed previously for diamine-appended Mg_2_(dobpdc) materials^34,37,42^ that may be due to stabilization of the CO_2_-adsorbed phase through hydrogen bonding with water.^34^ The overall gas uptake in ee-2–Mg_2_(olz) under humid CO_2_ is 19.6 g/100 g at 40 °C, slightly higher than that under dry conditions and reflecting the presence of co-adsorbed water (Figure 5a, red curve). When cooled under humid 15% CO_2_ in N_2_ (Figure 5a, blue curve), ee-2–Mg_2_(olz) again exhibits stepped gas adsorption but with a slightly lower onset temperature of 60 °C relative to that under dry and humid CO_2_ streams. Altogether, these results indicate that the capture of CO_2_ in ee-2–Mg_2_(olz) occurs readily under simulated coal flue gas conditions. Of note, ee-2–Mg_2_(olz) exhibits step-shaped CO_2_ adsorption at even lower CO_2_ concentrations of 10 and 5% in humid N_2_ (Figure S19). Following adsorption of humid 15% CO_2_ in N_2_, ee-2–Mg_2_(olz) can be fully regenerated upon heating to 85 °C under pure CO_2_ (Figure 5b).

**Figure 6:**
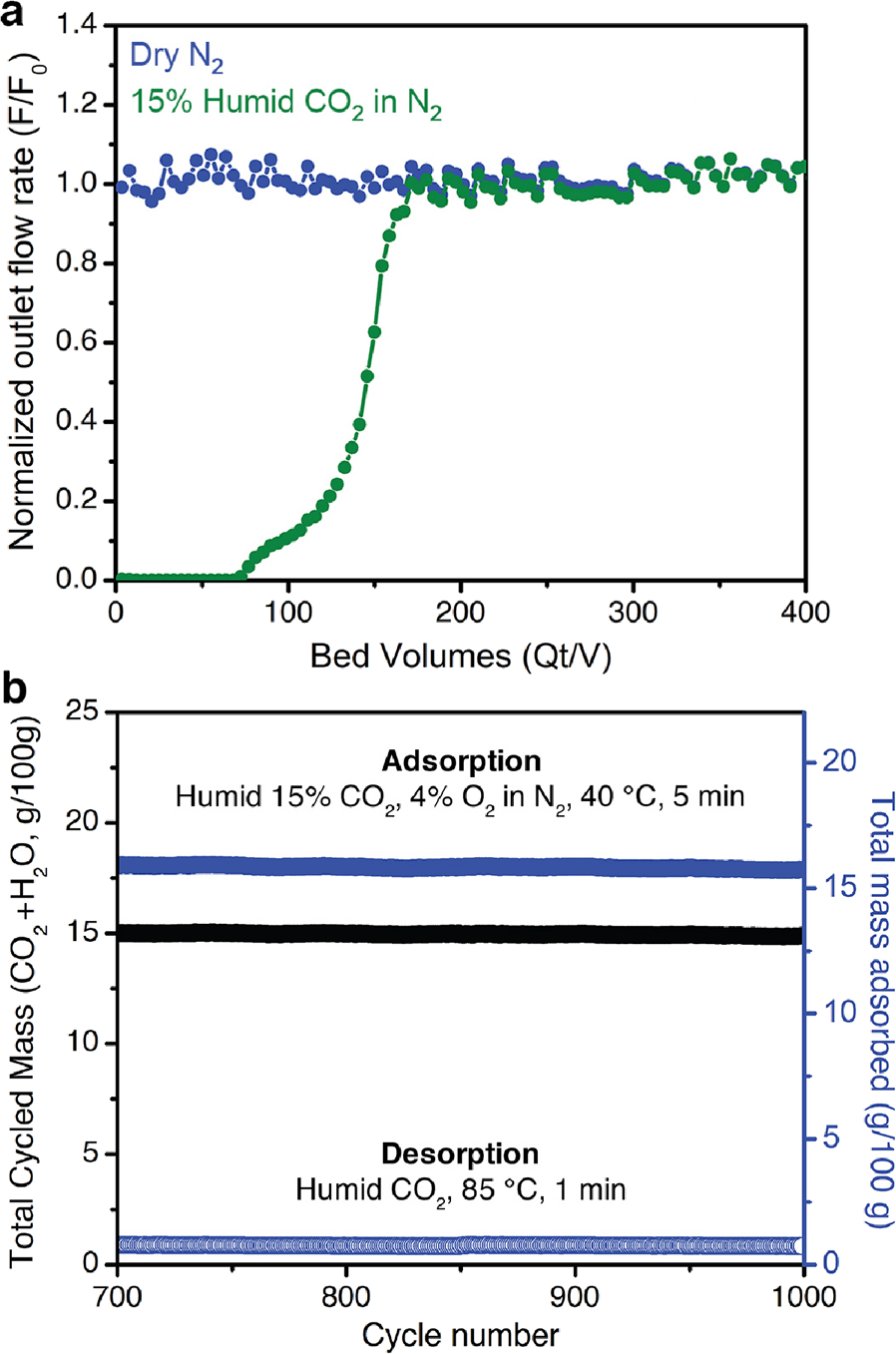
(a) Breakthrough data for ee-2–Mg_2_(olz) collected under humid (∼2.3% H_2_O) 15% CO_2_ in N_2_ at 40 °C with a flow rate of 10 sccm and ∼1 bar feed pressure. Breakthrough of N_2_ occurred nearly immediately, indicating negligible N_2_ uptake. The CO_2_ breakthrough profile exhibits a favorable sharp shape and corresponds to a total capacity of 3.9 ± 0.3 mmol/g. (b) Last 300 of 1000 thermogravimetric temperature-swing cycles conducted on ee-2–Mg_2_(olz) under simulated humid coal flue gas at atmospheric pressure. Adsorption, 40 °C, humid (∼2.3% H_2_O) 15% CO_2_, 4% O_2_ in N_2_, 5 min; desorption, 85 °C, humid (∼2.3% H_2_O) CO_2_, 1 min.

Breakthrough measurements were carried out to evaluate the performance of ee-2–Mg_2_(olz) under simulated coal flue gas CO_2_ capture conditions using a custom-built breakthrough apparatus (see Section 3 of the Supporting Information for details). In brief, a gram-scale fixed bed was filled with compressed, binder-free ee-2–Mg_2_(olz) pellets (∼350–700 μm diameter) and presaturated with water using a stream of humidified He gas. A gas stream containing humid 15% CO_2_ in N_2_ (∼2.3% H_2_O) was flowed through the column at 40 °C and atmospheric pressure, and the outlet composition and flow rate were tracked as a function of time. Rapid breakthrough of N_2_ occurred first, followed by breakthrough of CO_2_ much later (Figure 6a). From these data, we calculated a CO_2_ capacity of 3.9 ± 0.3 mmol/g from the humid, simulated flue gas stream, which corresponds to a 90% capture rate of 3.6 ± 0.3 mmol/g. Complete regeneration of the material was achieved by heating at 85 °C under a flow of humid He.

To probe the long-term stability of ee-2–Mg_2_(olz) to humid CO_2_ exposure under yet more realistic conditions, we evaluated the performance of ee-2–Mg_2_(olz) over the course of 1000 TGA adsorption (15% CO_2_, 4% O_2_, and ∼2.3% H_2_O in N_2_, 40 °C and 1 atm) and desorption (∼2.3% H_2_O in CO_2_ at 85 °C and 1 atm) cycles. Notably, the short adsorption (5 min) and desorption (1 min) intervals used throughout the course of the experiment highlight the rapid CO_2_/H_2_O adsorption and desorption kinetics in ee-2–Mg_2_(olz). After an initial equilibration period, the CO_2_/H_2_O cycling capacity remained relatively unchanged at ∼15.5 g/100 g during the last 300 cycles (Figure 6b; see also Figure S21). If the entirety of the adsorbed mass is presumed to be CO_2_, this capacity would correspond to an uptake of ∼3.52 mmol/g. Based on powder X-ray diffraction analysis and ^1^H NMR spectroscopy digestion experiments, respectively, ee-2–Mg_2_(olz) also retained crystallinity (Figure S22) and a high diamine loading of 99% after cycling, highlighting the robustness of the material to thermal and oxidative degradation in the presence of CO_2_, O_2_, and water. Overall, these data suggest that ee-2–Mg_2_(olz) is an exceptional candidate for CO_2_ capture from coal flue gas.

### Spectroscopic and Computational Analysis of CO_2_ Adsorption in Diamine–Mg_2_(olz)

Infrared (IR) spectroscopy, solid-state magic angle spinning NMR spectroscopy, and van der Waals (vdW)-corrected density functional theory (DFT) calculations were used to investigate the mechanism of cooperative CO_2_ adsorption in diamine–Mg_2_(olz), with ee-2–Mg_2_(olz) selected as the representative material. Diagnostic peaks for carbamate (ν(C–O) = 1630–1690 cm^–1^ and ν(C–N) = ∼1320 cm^–1^) were present in the IR spectrum collected for ee-2–Mg_2_(olz) dosed *in situ* with dry CO_2_ (Figure S15), supporting ammonium carbamate formation.

**Figure 7:**
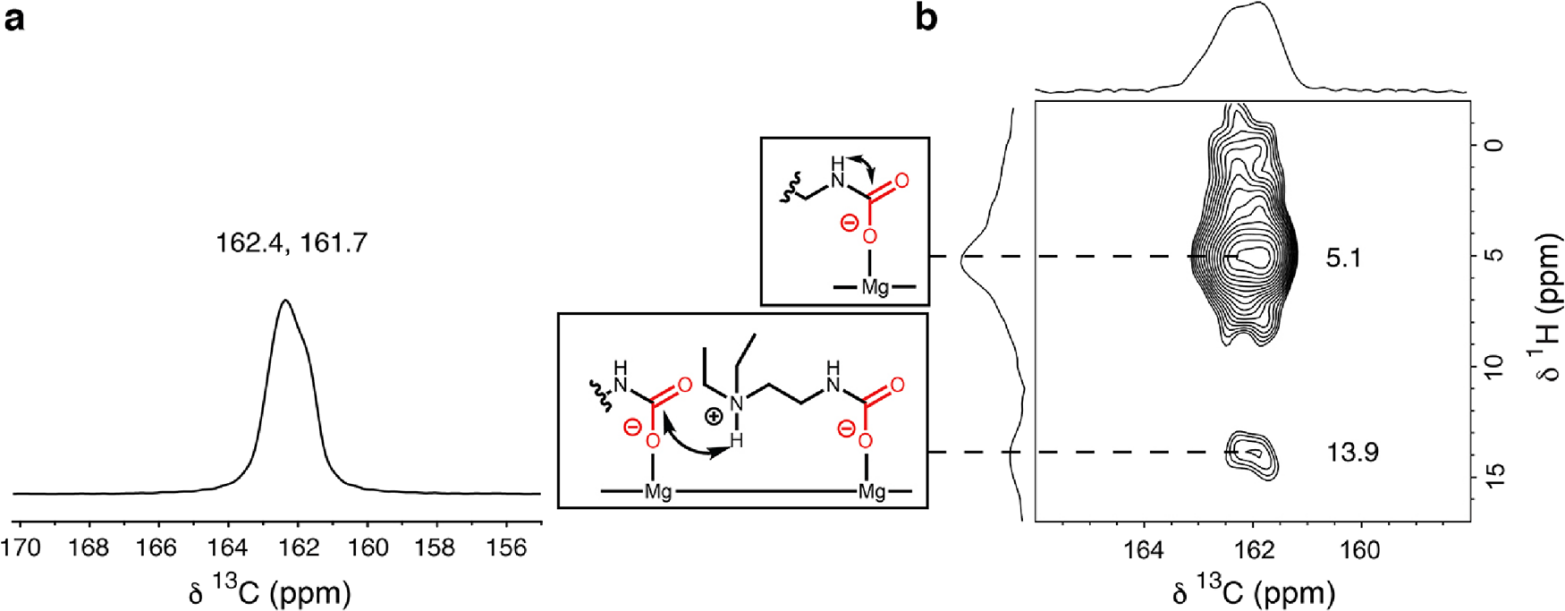
Room-temperature solid-state magic angle spinning NMR (16.4 T) spectra of ee-2–Mg_2_(olz) dosed with 1 bar ^13^CO_2_. (a) ^13^C NMR spectrum obtained by cross-polarization (with continuous-wave decoupling of ^1^H). (b) ^1^H → ^13^C HETCOR (contact time 100 μs) spectrum and correlation assignments.

The ^13^C NMR spectrum of ee-2–Mg_2_(olz) dosed with 1 bar of ^13^CO_2_ at room temperature features a resonance at 162.4 ppm, with a shoulder at approximately 161.7 ppm (Figure 7a). Both features were assigned to chemisorbed CO_2_ species and are consistent with those reported previously for carbamate formed in diamine-appended Mg_2_(dobpdc) upon CO_2_ uptake. For example, the resonance for the carbamate species formed upon CO_2_ adsorption in ee-2–Mg_2_(dobpdc) appears at 162.5 ppm.^50^ The presence of more than one resonance in the case of ee-2–Mg_2_(olz) is indicative of slightly different carbamate environments, which could arise because the larger pore size of Mg_2_(olz) can tolerate more conformations than Mg_2_(dobpdc). The two-dimensional (2D) ^1^H → ^13^C heteronuclear correlation (HETCOR) spectrum obtained for ^13^CO_2_-dosed ee-2–Mg_2_(olz) features strong correlations at 5.1 and 13.9 ppm, which are assigned to the presence of NHRCO_2_^–^ and NHR_3_^+^ species, respectively (Figure 7b),^50^ further supporting ammonium carbamate chain formation.^50^ We note that the 2D ^1^H → ^13^C HETCOR spectrum for ^13^CO_2_-dosed ee-2–Mg_2_(dobpdc) is very similar to correlations at 4.7 and 13.7 ppm; this result suggests similar hydrogen bond strengths in the ammonium carbamate chains formed in each compound (Figure S23).

**Figure 8:**
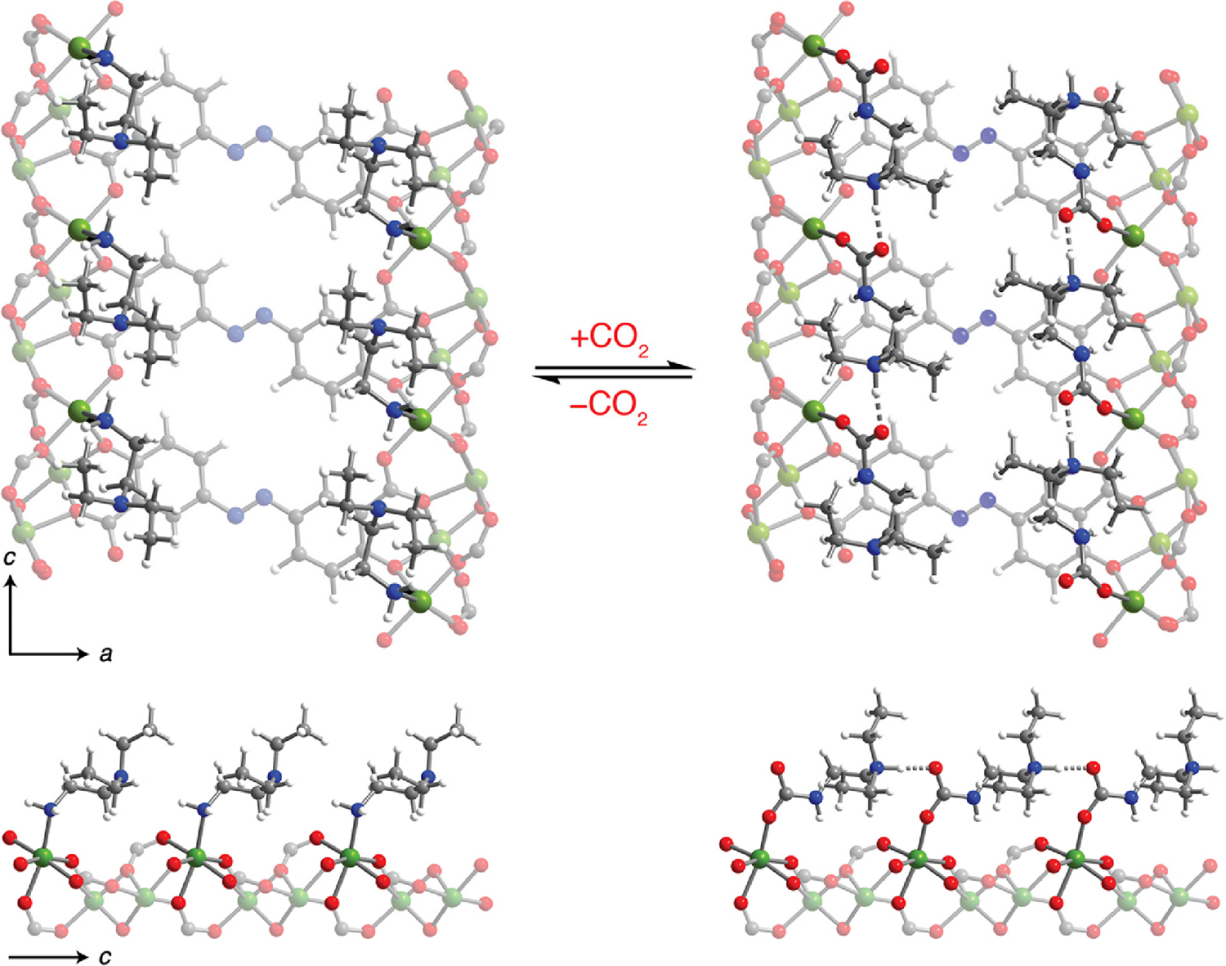
Proposed structures of (left) evacuated ee-2–Mg_2_(olz) and (right) ammonium carbamate chains formed upon the adsorption of CO_2_ in ee-2–Mg_2_(olz). Green, red, blue, gray, and white spheres represent Mg, O, N, C, and H, respectively.

To further confirm the ammonium carbamate chain formation mechanism, we used vdW-corrected DFT to simulate geometry-optimized structures for activated and fully CO_2_-dosed structures of ee-2–Mg_2_(olz) (Figure 8). Using these structures, CO_2_ binding energies (Δ*E*_ads_) and NMR chemical shifts were calculated to compare with the experimental values (Tables S5–S7). At full capacity, the calculated CO_2_ binding energy of ee-2–Mg_2_(olz) is −66.4 kJ/mol, which is comparable to the experimental differential adsorption enthalpy of −69.9 ± 0.8 kJ/mol. The framework e-2–Mg_2_(olz) was also modeled by using the same method, which yielded Δ*E*_ads_ = −88.6 kJ/mol. This energy agrees well with the experimental differential adsorption enthalpy of −87.1 ± 0.4 kJ/mol (see Figure S13 and Table S5) and supports ammonium carbamate formation as the mechanism of CO_2_ uptake in diamine–Mg_2_(olz). Analogous calculations were carried out for ee-2–Mg_2_(dobpdc) and e-2–Mg_2_(dobpdc), and the resulting CO_2_ binding energies are within ±5 kJ/mol of the corresponding experimental Δ*h*_ads_ values (Table S5).^50^ In addition, the calculated carbamate ^13^C NMR chemical shift in CO_2_–ee-2–Mg_2_(olz) at full capacity (i.e., one CO_2_ per diamine) is 164.8 ppm, close to the experimental value of 162.4 ppm (Table S7). Similarly, the calculated ammonium and carbamate ^1^H NMR shifts of 3.8 and 13.4 ppm, respectively, are in good agreement with the experiment (Table S7). Taken together, the spectroscopic and computational results support the formation of ammonium carbamate chains upon adsorption of CO_2_ in diamine-functionalized Mg_2_(olz) variants.

## Conclusions

We have developed a new class of robust cooperative CO_2_ adsorbents, diamine–Mg_2_(olz), that exhibit single-step CO_2_ uptake for various 1°,1°-, 1°,2°-, and 1°,3°-diamines. As a result, smaller temperature swings can be used to access the full CO_2_ capacity of these materials relative to isoreticular diamine–Mg_2_(dobpdc) frameworks that exhibit two-step CO_2_ adsorption. For diamine–Mg_2_(olz) compounds featuring diamines with branching alkyl substituents, both diamine sterics and the basicity of the pore-dwelling amine are important factors to consider in tuning the CO_2_ adsorption step pressure or temperature. Additionally, within a given class of diamines (e.g., 1°,3°-diamines), our results demonstrate that increasing the steric bulk of the pore-dwelling amine gives rise to less sharp CO_2_ uptake, indicating a lower degree of cooperativity in adsorption.

One variant studied here, ee-2–Mg_2_(olz), stands out as a particularly promising candidate for the capture of CO_2_ from coal flue gas. For example, the thermodynamics of CO_2_ adsorption in this material (Δ*h*_ads_ = −69.9 ± 0.8 kJ/mol and Δ*s*_ads_ = −198 ± 2 J/mol·K) are such that it can capture more than 90% of the CO_2_ present in a simulated coal flue gas stream (humid 15% CO_2_ in N_2_). Adsorption of CO_2_ at 40 °C and regeneration at 85 °C under 1 bar of CO_2_ under fixed-bed multicomponent conditions are associated with a high working capacity of 3.9 mmol/g and a low regeneration energy of 2.15 MJ/kg CO_2_. In addition, ee-2–Mg_2_(olz) also maintains excellent performance over the course of long-term cycling under simulated coal flue gas conditions with less than 1% diamine loss over 1000 cycles and a stable operating capacity of 15.5 g/100 g. Solid-state NMR spectroscopy and *in situ* IR spectroscopy data, supported by vdW-corrected DFT calculations, indicate that ee-2–Mg_2_(olz) captures CO_2_ via the formation of ammonium carbamate chains. Based on the promising performance of ee-2–Mg_2_(olz) for CO_2_ capture from simulated coal flue gas, we envision that other amine-functionalized Mg_2_(olz) materials can be readily optimized for CO_2_ removal from a variety of other process and emission streams.

## Experimental Section

### General Procedures

All synthetic manipulations were carried out in air, unless noted otherwise. All reagents and solvents were purchased from commercial suppliers at reagent-grade purity or higher and used without further purification. Custom gas blends of 15% (with and without 4% O_2_), 10%, 5%, and 1.5% CO_2_ in N_2_ were purchased from Praxair. The solution-phase ^1^H nuclear magnetic resonance (NMR) spectra of digested framework samples were collected on a Bruker AMX 300 or 400 MHz NMR spectrometer and referenced to residual dimethyl sulfoxide (δ = 2.50 ppm) or chloroform (δ = 7.26 ppm). The attenuated total reflectance IR spectra were collected on a PerkinElmer Spectrum 400 Fourier transform IR spectrometer. The linker H_4_olz was prepared following to the reported procedure.^44^

### Synthesis of Mg_2_(olz)

The framework Mg_2_(olz) was synthesized employing a modified version of the previously reported procedure.^44^ Using sonication, the H_4_olz ligand (11.40 g, 37.74 mmol) and Mg(NO_3_)_2_·6H_2_O (12.01 g, 47.10 mmol) were dissolved in a 55:45 (v:v) mixture of methanol and *N*,*N*-dimethylformamide (DMF) with a total volume of 200 mL. The solution was then filtered to remove any undissolved particles and transferred to a 350 mL glass pressure vessel with a Teflon-coated magnetic stir bar. The glass vessel was sealed with a Teflon cap and heated in a silicone oil bath at 120 °C for 20 h with stirring, after which time a yellow powder had formed. The crude product was vacuum filtered to isolate it from the synthesis solvent and subsequently soaked in 300 mL of DMF at 120 °C for a minimum of 3 h. The solid was again collected by vacuum filtration, and this soaking process was repeated two more times. After the final soak, the solid was isolated using vacuum filtration, and the powder was subsequently soaked in 300 mL of methanol at 60 °C for a minimum of 3 h. The methanol-soaked solid was isolated by vacuum filtration, and this soaking process was repeated two more times. After the final soak, the resulting yellow methanol-solvated material was isolated by using vacuum filtration and stored under fresh methanol when not in use. The methanol-solvated framework was desolvated under flowing N_2_ for 12 h at 180 °C to yield fully desolvated Mg_2_(olz) as a bright yellow powder. The powder X-ray diffraction pattern obtained for this material (Figure S1) and the calculated Langmuir surface area (77 K, N_2_) of 5070 m^2^/g (Figure S3) are consistent with that previously reported for Mg_2_(olz).^44^

### Synthesis of Diamine-Appended Mg_2_(olz) Compounds

Diamine–Mg_2_(olz) frameworks were prepared in a manner similar to that previously reported for diamine–Mg_2_(dobpdc).^34^ Methanol-solvated Mg_2_(olz) (∼150 mg) was filtered using a Büchner funnel and added to a 5 mL solution consisting of 20% (v:v) diamine in toluene. After being soaked for 14 h, the solid was filtered and washed three times with 10 mL of toluene to remove excess diamine in the framework pores prior to activation. The materials were then activated under flowing N_2_ for 30 min at temperatures ranging from 120 to 160 °C before analysis of their CO_2_ adsorption properties. The specific activation temperature for each material was determined based on the results of thermogravimetric decomposition analysis (Table S2). Diamine loadings were determined from analysis of the ^1^H NMR spectra collected for digested MOF samples, which were prepared by dissolving ∼2 mg of material in a solution containing 0.5 mL of dimethyl sulfoxide-*d*_6_ and 100 μL of deuterium chloride solution (35 g/100 g in D_2_O, ≥99 atom % D). Surface areas, powder X-ray diffraction patterns, decomposition profiles, IR spectra, and representative diamine loadings obtained from the ^1^H NMR spectra are presented in Figures S5–S7 and S15 and in Tables S1 and S2, respectively.

### Thermogravimetric Analysis

Dry TGA experiments were conducted using a TA Instruments TGA Q5000 or Discovery TGA, while humid CO_2_ TGA experiments were conducted using a TA Instruments TGA Q50 instrument where the inlet gas stream was humidified through two room-temperature water bubblers before entering the furnace. Masses were not corrected for buoyancy effects. Thermogravimetric decomposition experiments were carried out under 100% N_2_ with a temperature ramp rate of 2 °C/min from 30 to 600 °C (Figures S2 and S7). For isobaric measurements, samples were measured at ambient pressure by using a gas flow rate of 25 mL/min. Samples were first activated under flowing N_2_ at 120–160 °C for 30 min, after which time the temperature was rapidly increased to the highest plotted temperature for each adsorption (cooling) isobar. The gas was then switched to 100% CO_2_ (or a CO_2_/N_2_ blend) and held isothermally for 30 min to allow the gas to completely purge the system. Data were collected while cooling the sample to 30 °C at a rate of 1 °C/min. The material was then reheated to 130–170 °C at a rate of 1 °C/min. For ii-2–Mg_2_(olz) only, the sample was held isothermally for 1 h at 30 °C prior to switching from adsorption to desorption to allow for complete CO_2_ uptake due to the slow adsorption kinetics of the material. Adsorption isobar step temperatures were determined as the inflection points of the isobars based on the peak of the temperature derivative. Desorption isobar step temperatures were determined as the point of closure of the hysteresis loop.

For cycling experiments, a sample of ee-2–Mg_2_(olz) was first activated at 130 °C for 30 min under a flowing humid gas stream containing 15% of the dissolved CO_2_ in N_2_. The temperature was then rapidly decreased to 40 °C at a rate of 10 °C/min and held isothermally for 5 min. After the adsorption, the gas was switched to pure CO_2_ and the temperature was rapidly changed to 85 °C at a rate of 10 °C/min and held isothermally for 1 min. This adsorption–desorption process was repeated for 1000 cycles.

### Differential Scanning Calorimetry

DSC experiments were conducted using a TA Instruments Q200 DSC. Samples were analyzed under ambient pressure of He using a gas flow rate of 25 mL/min. Samples were activated at 130 °C under flowing N_2_ for 30 min to determine the activated sample mass before being transferred to the instrument and then reactivated at 130 °C under flowing He for 30 min prior to DSC measurements.

### Powder X-ray Diffraction Measurements

Laboratory powder X-ray diffraction patterns were collected on a Bruker AXS D8 Advance diffractometer using Cu *K*_α_ radiation (λ = 1.5418 Å), with samples placed on a zero-background sample holder. Synchrotron powder X-ray diffraction data were collected on Beamline 17-BM-B at the Advanced Photon Source at Argonne National Laboratory, with an average wavelength of λ = 0.45399 Å. desolvated samples were packed into borosilicate glass capillaries (1.0 mm in diameter) under a N_2_ atmosphere before being attached to a custom-designed gas-dosing cell equipped with a gas valve. These cells were then mounted on the goniometer head and connected to a gas-dosing manifold for *in situ* diffraction measurements. The sample temperature was controlled using an Oxford CryoSystems Cryostream 800. Samples were briefly heated to 120 °C under dynamic vacuum and then cooled to 298 K for data collection. The gas-dosing manifold was used to dose the framework with 1 bar of CO_2_ at 25 °C. Diffraction patterns were recorded using a PerkinElmer a-Si Flat Panel detector (Figure S6) and were monitored to confirm that the samples had reached equilibrium under gas-dosing conditions. Precise unit cell parameters were obtained using structureless Pawley refinements, which were performed in TOPAS-Academic 4.1^51^ (Tables S8 and S9).

### Gas Adsorption Isotherms

Carbon dioxide adsorption isotherms were collected on a Micromeritics 3Flex gas adsorption analyzer, and N_2_ adsorption isotherms were collected on a Micromeritics ASAP 2420 instrument. All gases were 99.998% purity or higher. The temperature was controlled by an oil bath or liquid nitrogen. Approximately 40–60 mg of diamine-functionalized MOF was added to a glass adsorption tube equipped with a Micromeritics *Transeal* for adsorption analysis. Samples were regenerated at 100 °C under dynamic vacuum (<10 μbar) for 6 h between isotherms. The isotherm data points were considered equilibrated after <0.01% pressure change occurred over 11 consecutive equilibration time intervals (15 s).

### Calculations of Differential Enthalpies and Entropies of Adsorption

The differential enthalpy (Δ*h*_ads_) of CO_2_ adsorption for each diamine–Mg_2_(olz) framework was calculated using the Clausius–Clapeyron relationship (eq 1).^48^
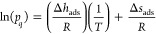
1

From the isotherm fits, the exact pressures (*p*_*q*_) corresponding to constant 1 mmol/g of CO_2_ loadings (*q*) were determined at different temperatures (*T*) by plotting ln(*p*_*q*_) versus 1/*T* at constant values of *q*. The *y*-intercepts of these linear trendlines are equal to – Δ*s*_ads_/*R* at each loading (with *p*_0_ = 1 bar), and the slopes were used to determine the corresponding differential enthalpies of adsorption. Further details are provided in Figures S13 and S14 and Table S5.

### Solid-State Magic Angle Spinning (MAS) ^13^C NMR Spectroscopy

A sample of ee-2–Mg_2_(olz) was activated under flowing N_2_ at 130 °C for 30 min and subsequently packed into a 3.2 mm zirconia NMR rotor inside a nitrogen-filled glove bag. The rotor was then evacuated inside a custom-built gas-dosing manifold for 10 min. Subsequently, ^13^CO_2_ gas (Sigma-Aldrich, 99 atom % ^13^C, <3 atom % ^18^O) was dosed into the sample, and a 30 min period was allowed for equilibration. The rotor was then capped inside the manifold using a moveable plunger (see our previous work for details on this apparatus^50^). The final ^13^CO_2_ pressure was 1 bar. Dosing was performed at room temperature.

Solid-state NMR experiments were performed at 16.4 T by using a 3.2 mm Bruker MAS probe. A MAS rate of 15 kHz was used for all experiments. The ^13^C NMR spectra were acquired by cross-polarization from ^1^H with a contact time of 1 ms and with continuous-wave decoupling during acquisition. The 2D HETCOR experiments also employed magnetization transfer by cross-polarization with a short contact time of 100 μs used to selectively show short-range correlations. The ^1^H and ^13^C chemical shifts were referenced to 1.8 ppm (adamantane) and 38.5 ppm (adamantane, tertiary carbon–left-hand resonance), respectively.

## Supporting Information Available

Crystallographic data for olsalazine (ZIP)
Additional full experimental characterization data and computational details (PDF)
