# Supplementary material for: Cooperative Carbon Dioxide Capture in Diamine-Appended Magnesium–Olsalazine Frameworks
Source: J Am Chem Soc. 2023 Jul 26;145(31):17151–63. doi: 10.1021/jacs.3c03870 (PMC10416307; doi:10.1021/jacs.3c03870)
Supplement: Supplementary file 2 — ja3c03870_si_003.pdf [file ja3c03870_si_003.pdf]

Supporting information for:

# Cooperative Carbon Dioxide Capture in Diamine–Appended Magnesium–Olsalazine Frameworks

Ziting Zhu,[1,3,5] Surya T. Parker,[2,5] Alexander C. Forse,[2,3,⊥] Jung-Hoon Lee,[4,6,¶] Rebecca L. Siegelman,[3,5,†] Phillip J. Milner,[3,5,‡] Hsinhan Tsai,[3,5] Mengshan Ye,[3] Shuoyan Xiong,[3] Maria V. Paley,[3] Adam A. Uliana,[2,5] Julia Oktawiec,[3] Bhavish Dinakar,[2,5] Stephanie A. Didas,[5] Katie R. Meihaus,[3] Jeffrey A. Reimer,[2,5] Jeffrey B. Neaton,[4,6] Jeffrey R. Long*[2,3,5]

[1]Department of Materials Science and Engineering, [2]Department of Chemical and Biomolecular Engineering, [3]Department of Chemistry, [4]Department of Physics, University of California, Berkeley, California 94720, United States

[5]Materials Sciences Division, Lawrence Berkeley National Laboratory, Berkeley, California 94720, United States

[6]Molecular Foundry, Lawrence Berkeley National Laboratory, Berkeley, California 94720, United States

*Correspondence to Jeffrey R. Long: jrlong@berkeley.edu

**Present Addresses:**

[⊥]Department of Chemistry, University of Cambridge, Cambridge, CB2 1EW, United Kingdom

[¶]Computational Science Research Center, Korea Institute of Science and Technology (KIST), Seoul 02792, Republic of Korea

[†]DuPont de Nemours, Inc. 200 Powder Mill Rd, Wilmington, DE, 19803, United States

[‡]Department of Chemistry and Chemical Biology, Cornell University, Ithaca, New York 14853, United States

**Table of Contents**


1. **$Mg_2$(olz) Synthesis and Characterization** .......... S3
   - Powder X-Ray Diffraction Data .......... S3
   - Thermogravimetric Decomposition Data .......... S3
   - $N_2$ Adsorption Isotherms and Surface Area Calculations .......... S4
   - Scanning Electron Microscopy of $Mg_2$(olz) and ee-2–$Mg_2$(olz) Crystallites .......... S5
2. **Diamine–$Mg_2$(olz) Characterization** .......... S6
   - $N_2$ Adsorption Isotherms and Surface Area Calculations .......... S6
   - Powder X-Ray Diffraction Data Before and After $CO_2$ Dosing .......... S8
   - Thermogravimetric Decomposition Data .......... S9
   - Diamine Loadings from $^1H$ NMR Spectroscopy and Activation Temperatures .......... S10
   - $CO_2$ Adsorption and Desorption Isobars .......... S11
   - $CO_2$ Adsorption Isotherms .......... S14
   - Hill Coefficients .......... S17
   - $CO_2$ Differential Enthalpies and Entropies .......... S19
   - Infrared Spectra of Diamine-Appended Metal–Organic Frameworks .......... S22
3. **Characterization of ee-2–$Mg_2$(olz)** .......... S23
   - $O_2$ and $N_2$ Adsorption Isobar and Isotherms .......... S23
   - $CO_2$ Adsorption Isobars in Different Concentrations .......... S25
   - Calculation of the Approximate Regeneration Energy of ee-2–$Mg_2$(olz) .......... S25
   - Adsorption/Desorption Cycling of ee-2–$Mg_2$(olz) .......... S26
   - Breakthrough Experiment Details .......... S27
   - Additional Solid-State Magic Angle Spinning $^{13}C$ NMR Spectra and Details .......... S29
   - Van der Waals-Corrected DFT Calculations .......... S30
   - Crystallographic Data .......... S32

## 1. $Mg_2$(olz) Synthesis and Characterization

### Powder X-Ray Diffraction

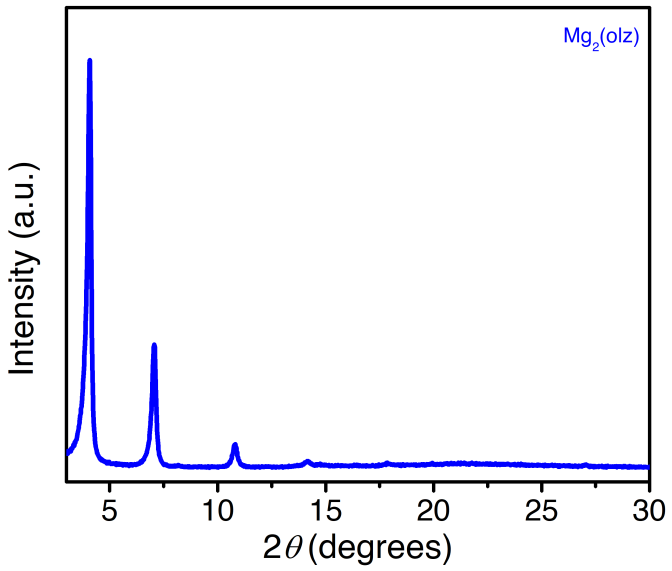


**Figure S1.** Laboratory powder X-ray diffraction pattern of $Mg_2$(olz) (CuK$\alpha$ radiation, $\lambda$ = 1.5418 Å).

### Thermogravimetric Decomposition Data

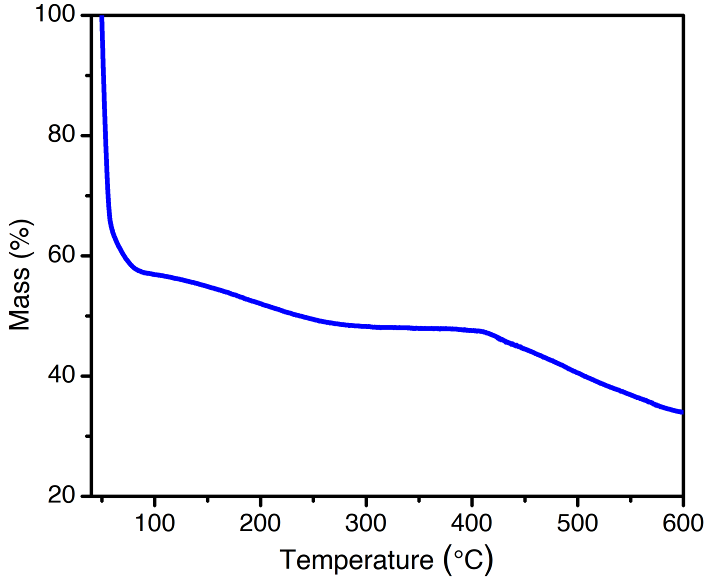


**Figure S2.** Dry $N_2$ decomposition profiles of $Mg_2$(olz). A ramp rate of 2 °C/min was used.

## $N_2$ Adsorption Isotherms and Surface Area Calculations

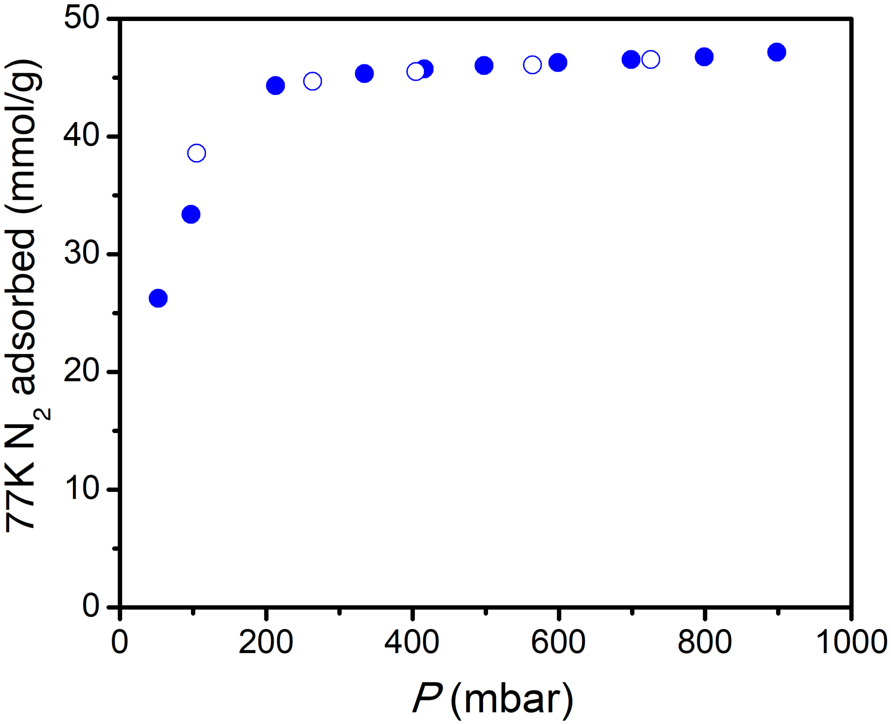


**Figure S3.** 77 K $N_2$ adsorption (solid) and desorption (open circles) isotherms of activated $Mg_2$(olz). The calculated Langmuir surface area is 5066.2 $m^2$/g.

**Scanning Electron Microscopy of $Mg_2$(olz) and ee-2–$Mg_2$(olz) Crystallites**

| | |
|---|---|
| **$Mg_2$(olz)** | 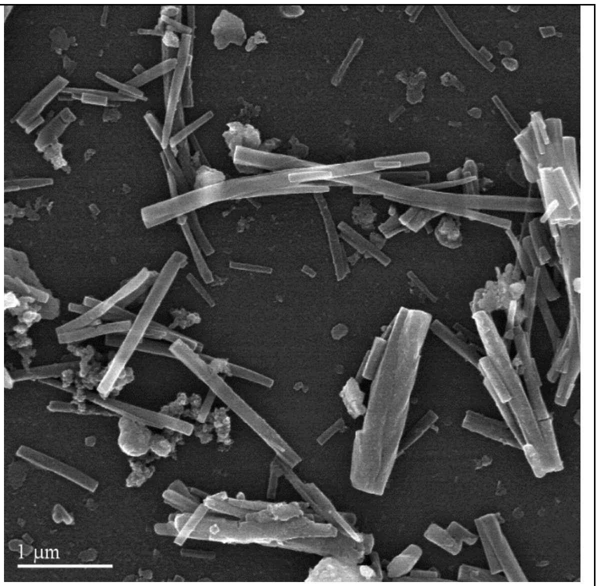  |
| **ee-2–$Mg_2$(olz)** | 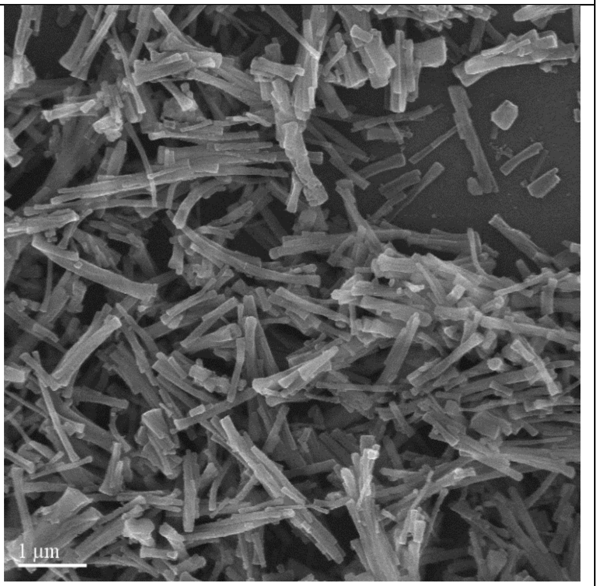  |

**Figure S4.** Scanning electron microscopy images of $Mg_2$(olz) and ee-2–$Mg_2$(olz) crystallites. Images were taken using a Hitachi S-5000 SEM at the Electron Microscope Laboratory at the University of California, Berkeley. Samples were dispersed in methanol and then drop casted onto silicon chips. To dissipate charge, samples were sputter coated with approximately 3 nm of gold (Tousimis). Scale bars: 1 μm.

## 2. Diamine–$Mg_2$(olz) Characterization

### $N_2$ Adsorption Isotherms and Surface Area Calculations

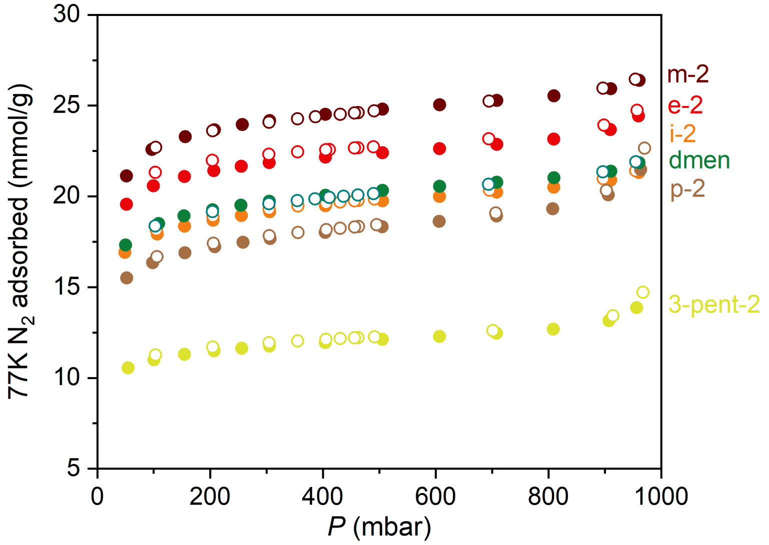


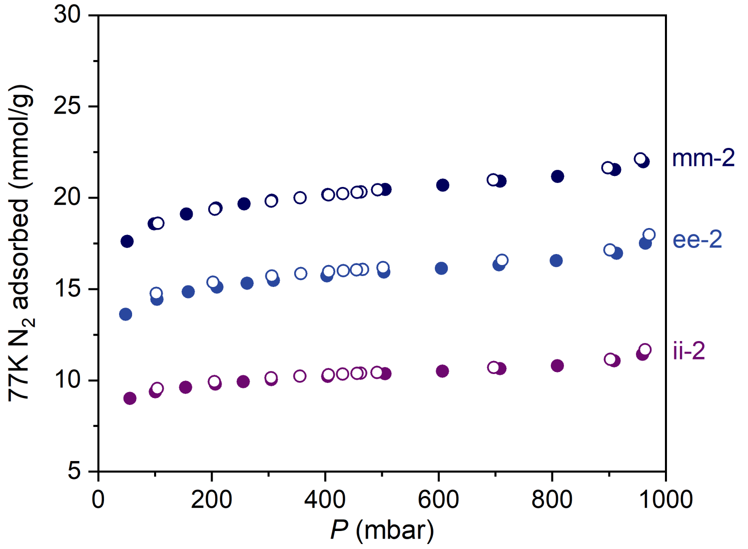


**Figure S5.** Nitrogen adsorption (solid) and desorption (open circles) isotherms colleted at 77 K for activated m-2–, e-2–, i-2–, dmen–, p-2–, and 3-pent-2–$Mg_2$(olz) (upper) and activated nm-2–, ee-2–, ii-2–$Mg_2$(olz) (lower).

**Table S1.** Surface areas for diamine-appended $Mg_2$(olz) analogues.

| Framework | Surface area (Langmuir) ($m^2$/g) |
| --- | --- |
| m-2–$Mg_2$(olz) | 2428.7 |
| e-2–$Mg_2$(olz) | 2185.2 |
| p-2–$Mg_2$(olz) | 1777.2 |
| i-2–$Mg_2$(olz) | 1919.5 |
| 3-pent-2–$Mg_2$(olz) | 1176.8 |
| dmen–$Mg_2$(olz) | 1980.5 |
| ee-2–$Mg_2$(olz) | 1550.9 |
| mm-2–$Mg_2$(olz) | 1989.6 |
| ii-2–$Mg_2$(olz) | 1006.1 |

## Powder X-Ray Diffraction Data Before and After $CO_2$ Dosing

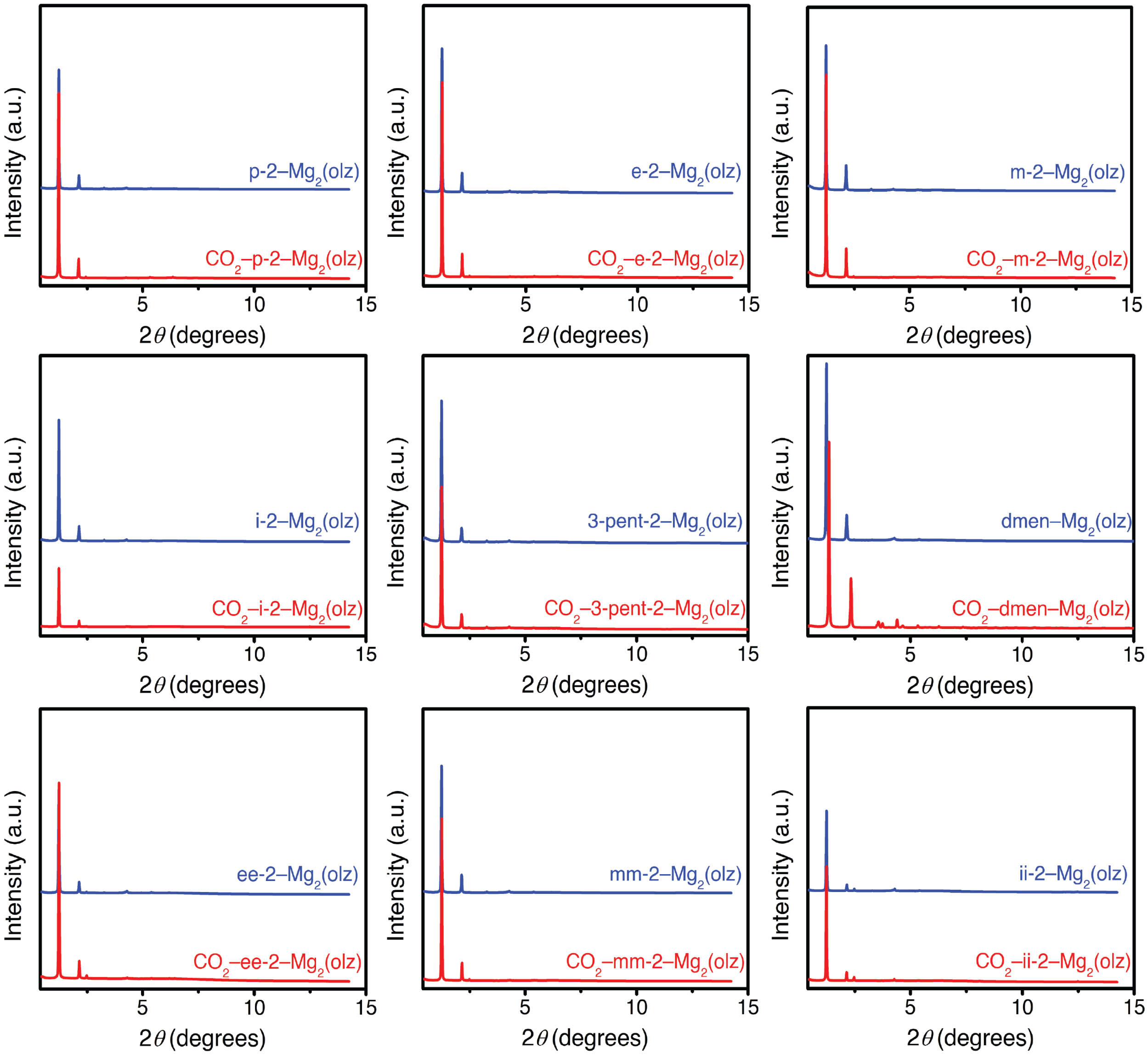


**Figure S6.** Powder x-ray diffraction patterns of diamine–$Mg_2$(olz) materials before and after *in situ* $CO_2$ dosing ($\lambda$ = 0.45399 Å). The materials were dosed under 1 bar $CO_2$ at room temperature. For all materials, the diffraction patterns are the same before and after $CO_2$ dosing, indicating the materials remain crystalline and retain their underlying structure during $CO_2$ capture.

## Thermogravimetric Decomposition Data

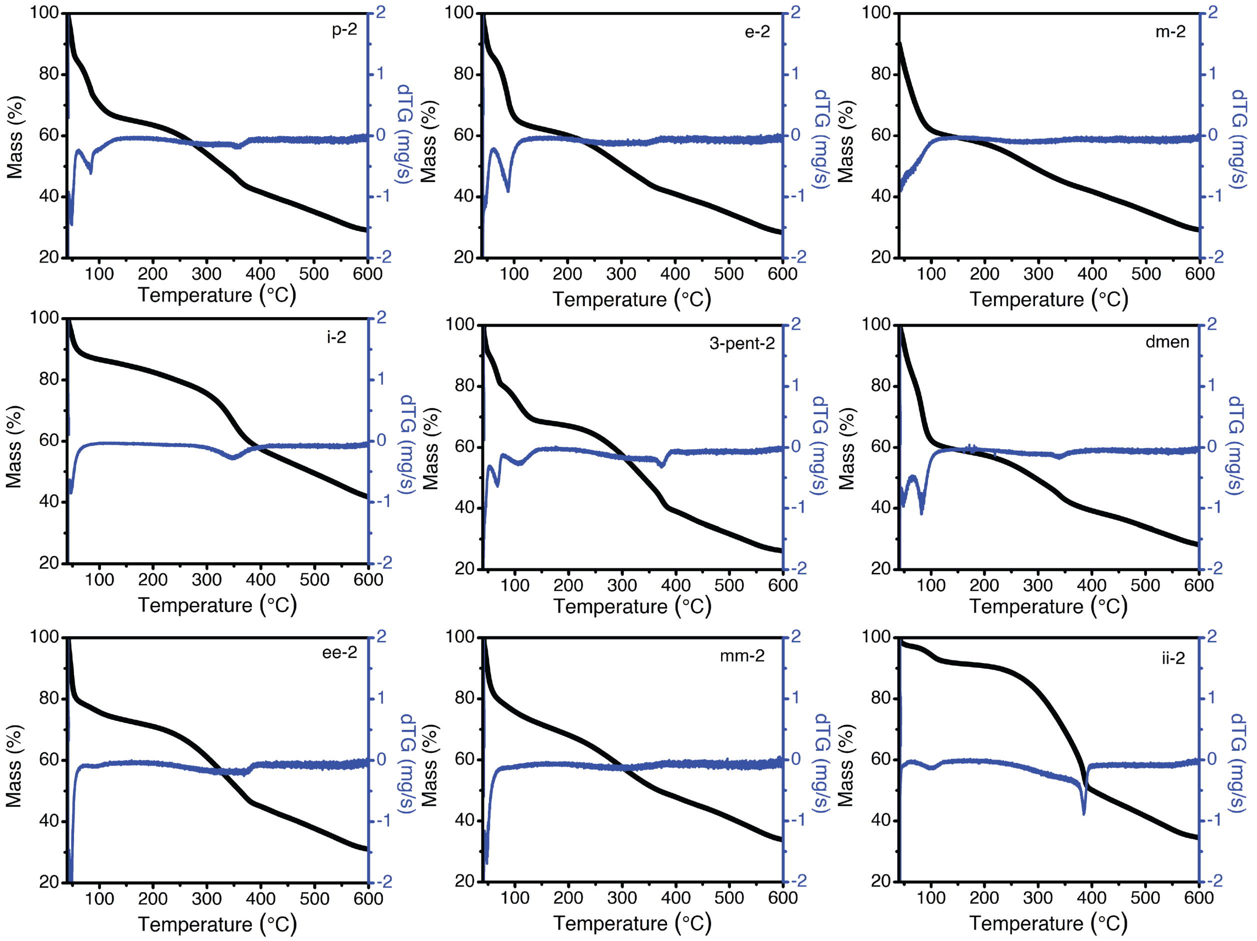


**Figure S7.** Dry $N_2$ decomposition profiles for diamine–$Mg_2$(olz) analogues. The thermogravimetric decomposition traces are marked in black while the derivative decomposition traces (dTG) are marked in blue. The lower temperature mass loss (<80 °C) corresponds to weakly absorbed species (e.g., $CO_2$, $H_2O$, toluene, hexane), the middle temperature mass loss (~100 °C) corresponds to excess diamines in the pore, and the higher temperature mass loss (>200 °C) corresponds to the metal-bound diamines. A ramp rate of 2 °C/min was used.

**Diamine Loadings from $^1$H NMR Spectroscopy and Activation Temperatures**

**Table S2.** Representative diamine loadings for diamine-appended $Mg_2$(olz) analogues calculated from the ratio of diamine peak integral(s) to ligand peak integrals by $^1$H NMR following sample digestion with DCl in DMSO-$d_6$. Material activation temperatures as determined by TGA analysis are also included.

| Framework | As-synthesized diamine loading (%) | Diamine loading after activation (%) | Activation Temperature (°C) | Diamine loading after adsorption/desorption cycling (%) |
|---|---|---|---|---|
| m-2–$Mg_2$(olz) | 177 | 99 | 160 | - |
| e-2–$Mg_2$(olz) | 206 | 106 | 150 | - |
| p-2–$Mg_2$(olz) | 166 | 103 | 150 | - |
| i-2–$Mg_2$(olz) | 176 | 104 | 150 | - |
| 3-pent-2–$Mg_2$(olz) | 116 | 104 | 130 | - |
| dmen–$Mg_2$(olz) | 122 | 101 | 120 | - |
| ee-2–$Mg_2$(olz) | 120 | 102 | 120 | 99 |
| mm-2–$Mg_2$(olz) | 140 | 102 | 120 | - |
| ii-2–$Mg_2$(olz) | 125 | 100 | 120 | - |

**Table S3.** Diamines used in this work to prepare diamine–$Mg_2$(olz) frameworks and the abbreviation used for each. The primary amine in each case (and the least sterically hindered primary amine in dmen) was presumed to bind to the framework $Mg^{2+}$ sites as discussed in the main text. To evaluate any correlation between the basicity of the pore-dwelling amine and the step pressure/temperature for $CO_2$ uptake, a representative structure was first generated by replacing the metal-bound primary amine in each case with a proton, see the structures in column 4 below. Experimental p$K_a$ values for the corresponding monoammonium cations are reported when available (in parentheses),[1] and in all cases p$K_a$ values were also calculated using SciFinder.[2]

| Diamine Name | Structure | Abbreviation | Structure used for conjugate acid p$K_a$ determination | p$K_a$ Calc (Exp) |
|---|---|---|---|---|
| *N,N*-diisopropyl ethylenediamine | 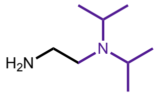 | ii-2 | 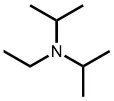 | 11.0 ± 0.3 |
| *N,N*–dimethyl ethylenediamine | 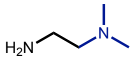 | mm-2 | 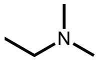 | 9.8 ± 0.3 (9.99) |
| *N,N*–diethyl ethylenediamine | 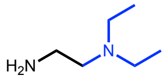 | ee-2 | 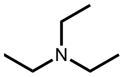 | 10.6 ± 0.3 (10.65) |
| 1,2-diamino-2-methylpropane | 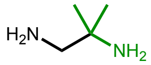 | dmen | 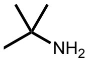 | 10.7 ± 0.3 (10.45) |
| *N*–(3-pentyl) ethylenediamine | 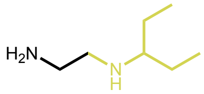 | 3-pent-2 | 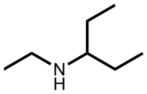 | 11.1 ± 0.1 |
| *N*–isopropyl ethylenediamine | 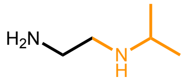 | i-2 | 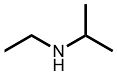 | 10.8 ± 0.2 |
| *N*–propyl ethylenediamine | 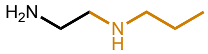 | p-2 | 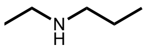 | 10.8 ± 0.2 (11.00 di-*n*-propyl amine) |
| *N*–ethyl ethylenediamine | 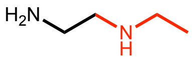 | e-2 | 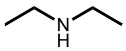 | 10.8 ± 0.1 (10.98) |
| *N*–methyl ethylenediamine | 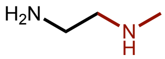 | m-2 | 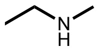 | 10.8 ± 0.1 (10.64 for dimethylamine) |

## $CO_2$ Adsorption and Desorption Isobars

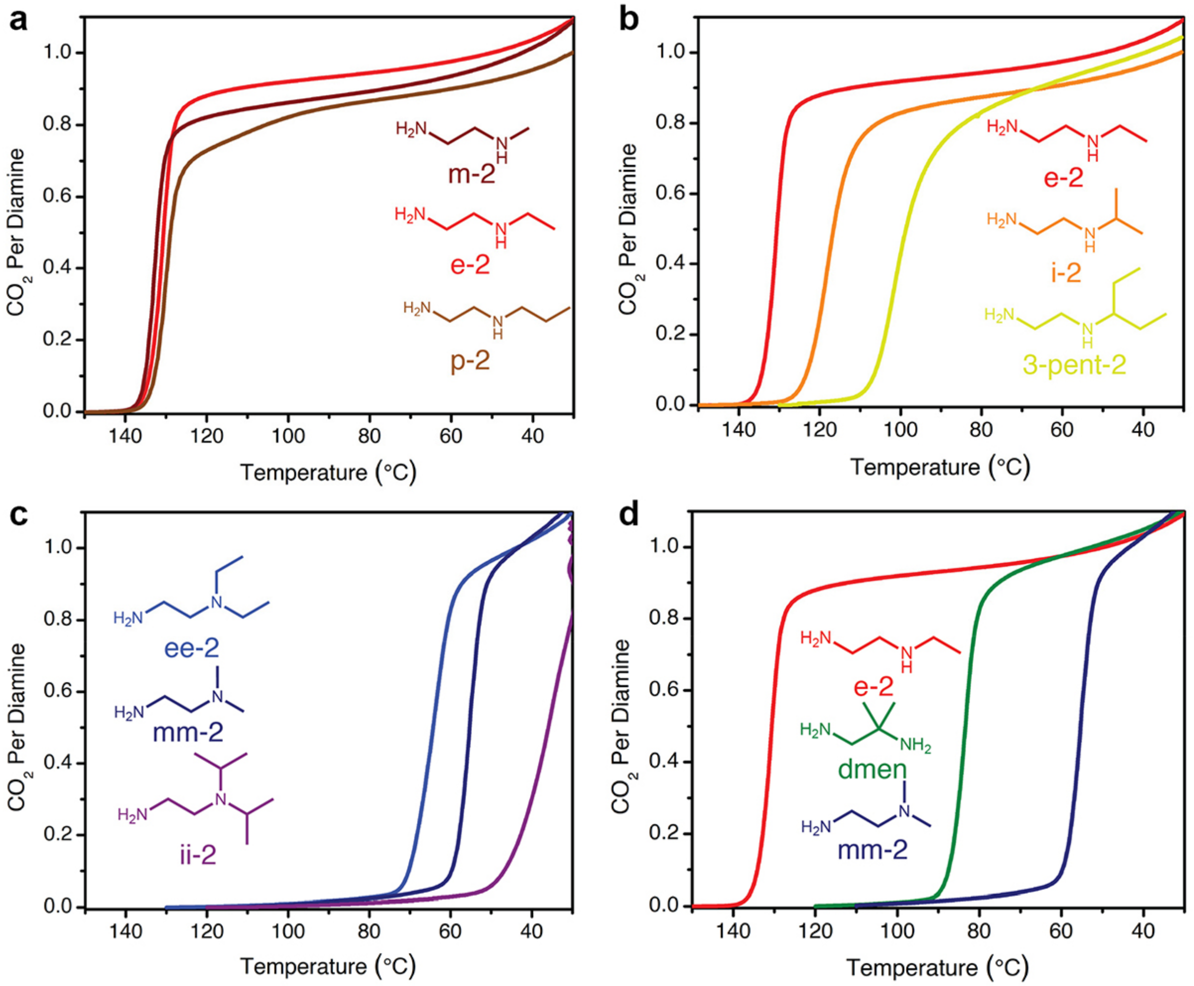


**Figure S8.** Adsorption isobars under pure $CO_2$ for a series of diamine–$Mg_2$(olz) variants bearing (a) 1°,2°-diamines with linear alkyl chains of different lengths, (b) 1°,2°-diamines with alkyl chains with different sizes, (c) 1°,3°-diamines with different degrees of branching on the tertiary amine, and (d) a comparison of a 1°,1°-, 1°,2°-, and 1°,3°-diamines with alkyl substitutions with the same total number of carbons.

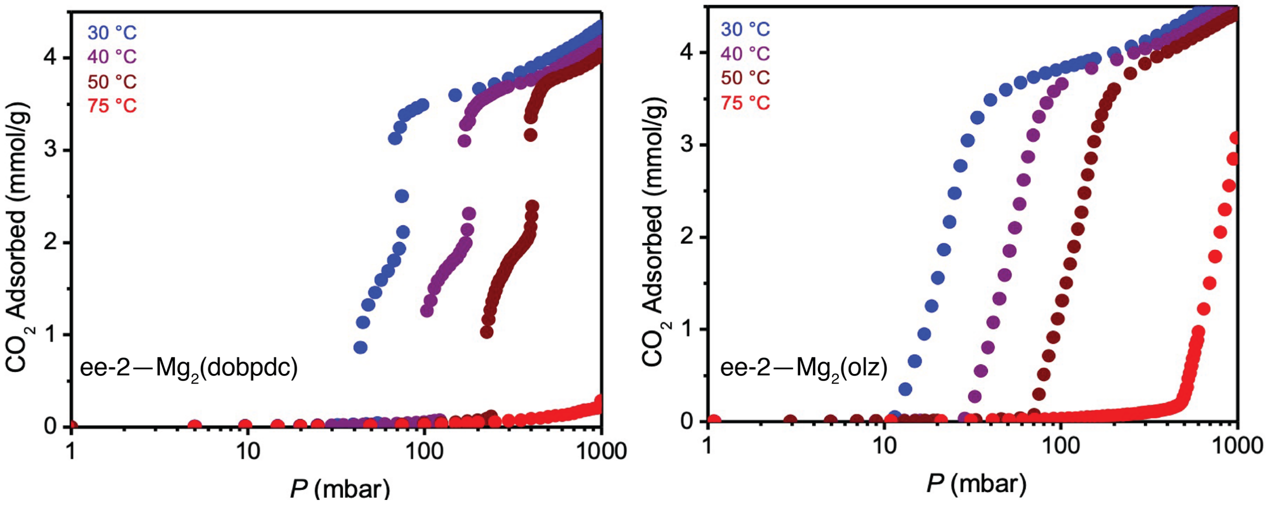


**Figure S9.** $CO_2$ adsorption isotherms at 30, 40, 50, and 75 °C for ee-2–$Mg_2$(dobpdc) (left) and ee-2–$Mg_2$(olz) (right). The change of framework from $Mg_2$(dobpdc) to $Mg_2$(olz) removes the double step, and the step positions also shift to lower pressures.

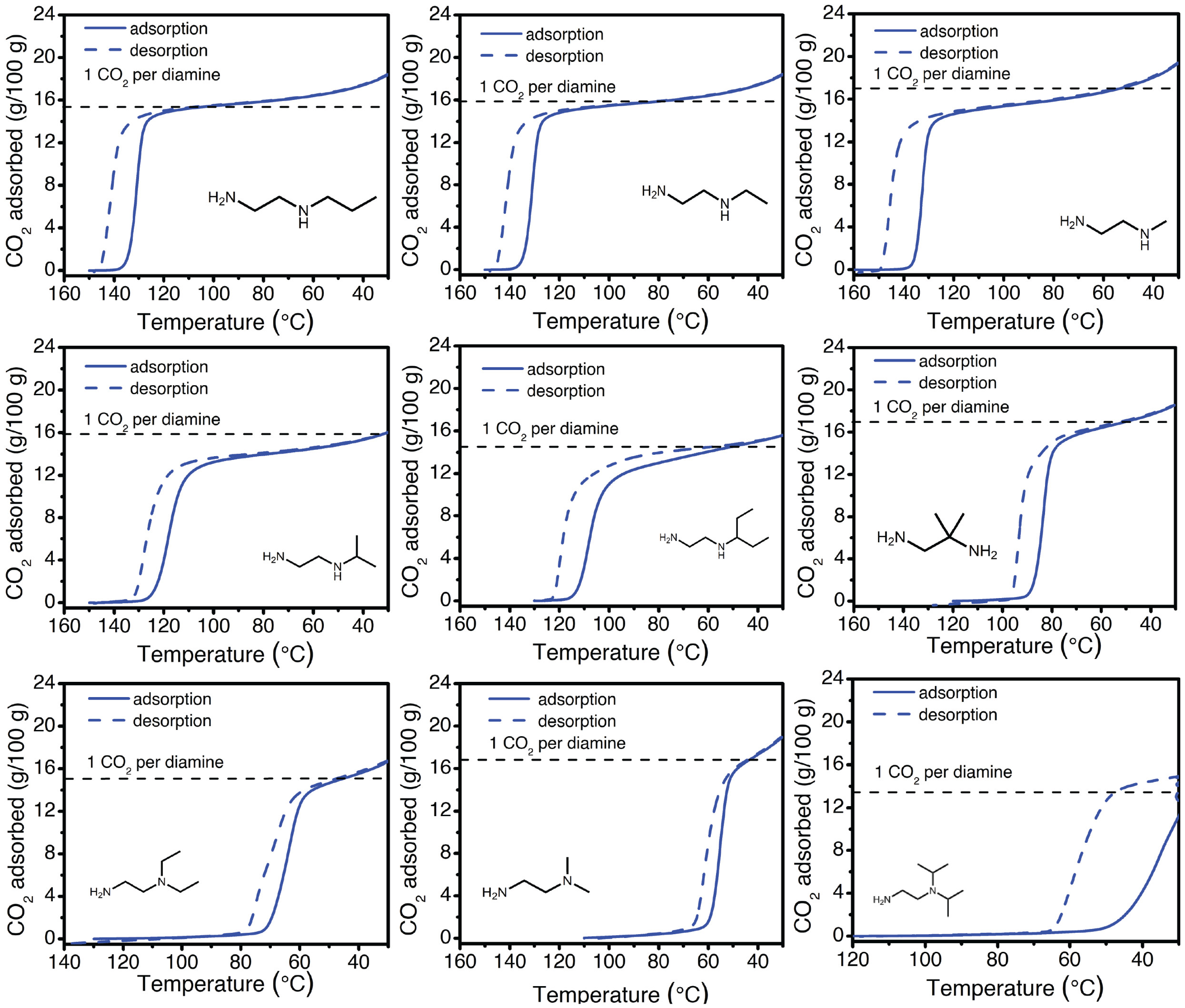


**Figure S10.** Dry $CO_2$ adsorption and desorption isobars for diamine–$Mg_2$(olz) at atmospheric pressure. The horizontal dashed line indicates the theoretical capacity for adsorption of 1 $CO_2$ per diamine, and the structure of the appended diamine is depicted at the bottom left. A ramp rate of 1 °C/min was used.

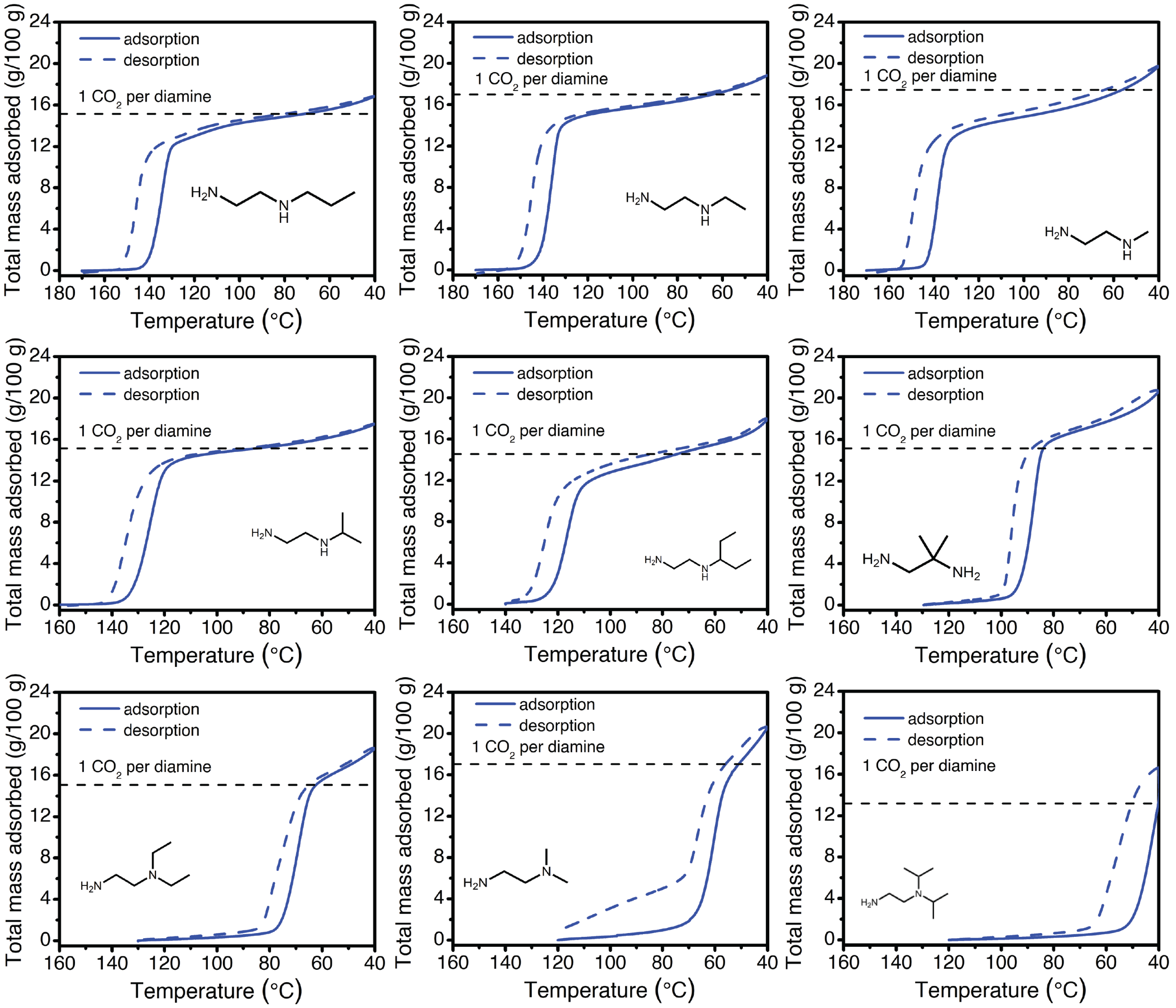


**Figure S11.** Humid (~1.5% $H_2O$) $CO_2$ adsorption and desorption isobars for diamine–$Mg_2$(olz) obtained at atmospheric pressure. The horizontal dashed line indicates the theoretical capacity for adsorption of 1 $CO_2$ per diamine, and the structure of the appended diamine is depicted at the bottom. A ramp rate of 1 °C/min was used.

## $CO_2$ Adsorption Isotherms

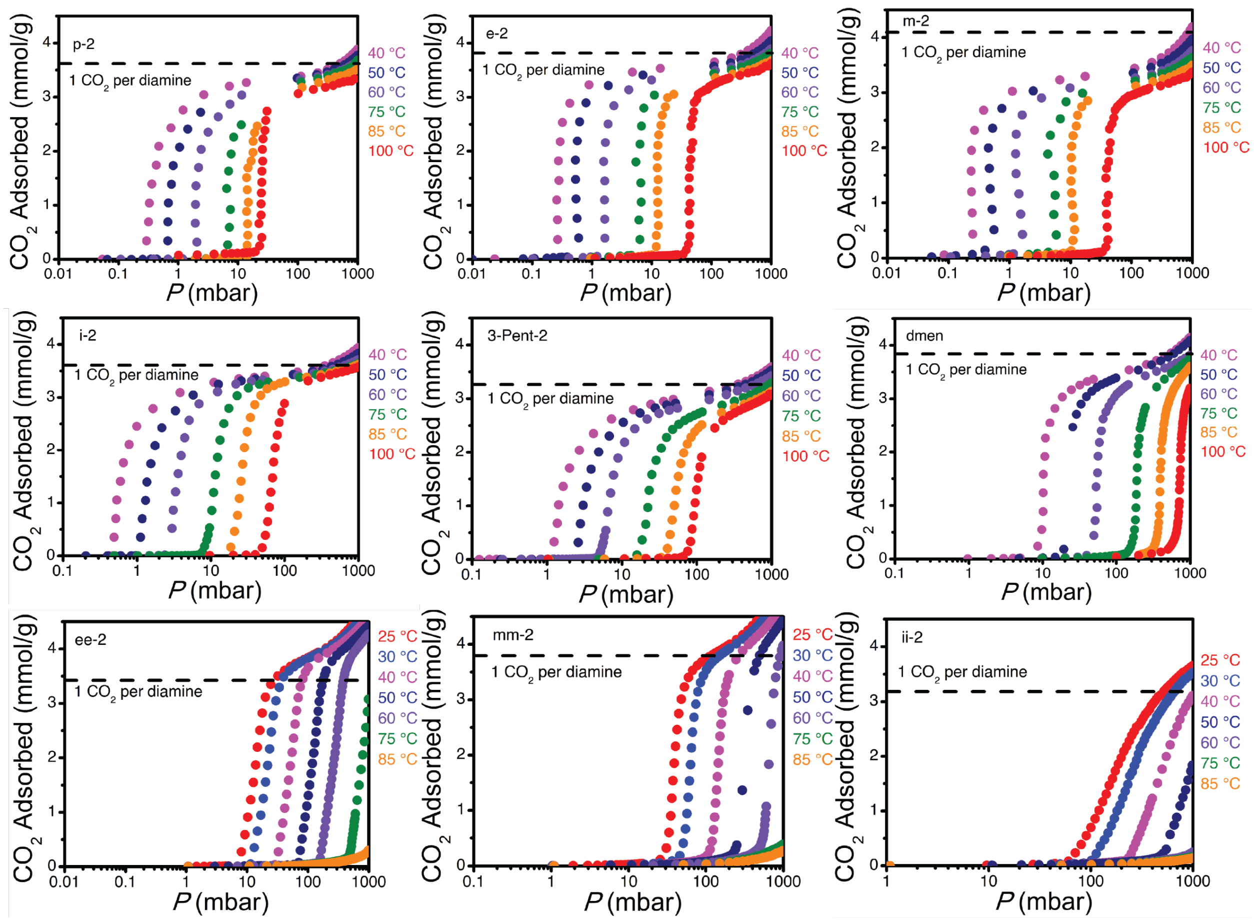


**Figure S12.** $CO_2$ adsorption isotherms at 25, 30, 40, 50, 60, 75, 85, and 100 °C for diamine–$Mg_2$(olz). Each sample was activated under flowing $N_2$ at 150 °C for 6 h, followed by activation under high vacuum (<10 μbar) at 100 °C for 4 h. The samples were reactivated under high vacuum (<10 μbar) at 100 °C for 6 h between isotherms.

**Hill Coefficients**

* The Hill equation is as follows[3]:

$$\theta = \frac{\frac{[L]^n}{K_d}}{1+\frac{[L]^n}{K_d}} = \frac{1}{1+\frac{K_d}{[L]^n}} = \frac{1}{1+[\frac{K_{0.5}}{L}]^n} = \frac{[L]^n}{[K_{0.5}]^n+[L]^n},$$

where $\theta$ is the fraction of the bound to total receptors, $[L]$ is the total ligand concentration, $K_d$ is the dissociation constant, $n$ is the Hill coefficient, and $K_{0.5}$ is the ligand concentration at which half of the receptors are bound and equivalent to the $n$th root of $K_d$. In our analysis, we used the following equation:

$$\theta_{CO_2} = \frac{[P]^n}{[P_{50\%}]^n+[P]^n}.$$

Here, $\theta_{CO_2}$ is defined as the fraction of bound $CO_2$ to the total capacity at the top of the adsorption step (in order to exclude physisorbed $CO_2$, which is reflected in the gradual $CO_2$ uptake following the adsorption step); $P$ is the $CO_2$ pressure; $P_{50\%}$ is the pressure when 50% of the diamines in the material have bound $CO_2$ (based on the diamine loadings determined via $^1$H NMR spectroscopy); and $n$ is the Hill coefficient. The fits were performed using MATLAB.[4]

**Table S4. Hill coefficients (*n*) for diamine–$Mg_2$(olz) at different temperatures.**

| *Framework* | 25°C | 30 °C | 40 °C | 50 °C | 60 °C | 75 °C | 85 °C | 100 °C | Avg |
|---|---|---|---|---|---|---|---|---|---|
| p-2–$Mg_2$(olz) | — | — | — | — | 5±1 | 6±2 | 6.6±0.8 | — | 6±2 |
| e-2–$Mg_2$(olz) | — | — | — | — | 10±3 | 10±3 | 12±1 | — | 11±4 |
| m-2–$Mg_2$(olz) | — | — | — | — | 13±4 | — | 10±1 | 10.7±0.7 | 11±4 |
| i-2–$Mg_2$(olz) | — | — | — | — | 6±1 | 8.1±0.1 | 7.6±0.2 | — | 7±1 |
| 3-pent-2–$Mg_2$(olz) | — | — | — | — | 7.3±0.6 | 6±2 | 5.4±0.9 | — | 6±2 |
| dmen–$Mg_2$(olz) | — | — | — | 14±1 | 12.2±0.6 | 13±2 | 12±1 | — | 13±3 |
| ee-2–$Mg_2$(olz) | 4.2±0.2 | 5.1±0.2 | 5.3±0.4 | 5.1 ± 0.3 | — | — | — | — | 4.9±0.6 |
| mm-2–$Mg_2$(olz) | 7.6±0.1 | 9.3±0.7 | 9.9±0.5 | 8.8± 0.8 | — | — | — | — | 9±1 |
| ii-2–$Mg_2$(olz) | 2.7±0.1 | 2.59±0.01 | 3.7±0.1 | — | — | — | — | — | 3.0±0.1 |

## $CO_2$ Differential Enthalpies and Entropies

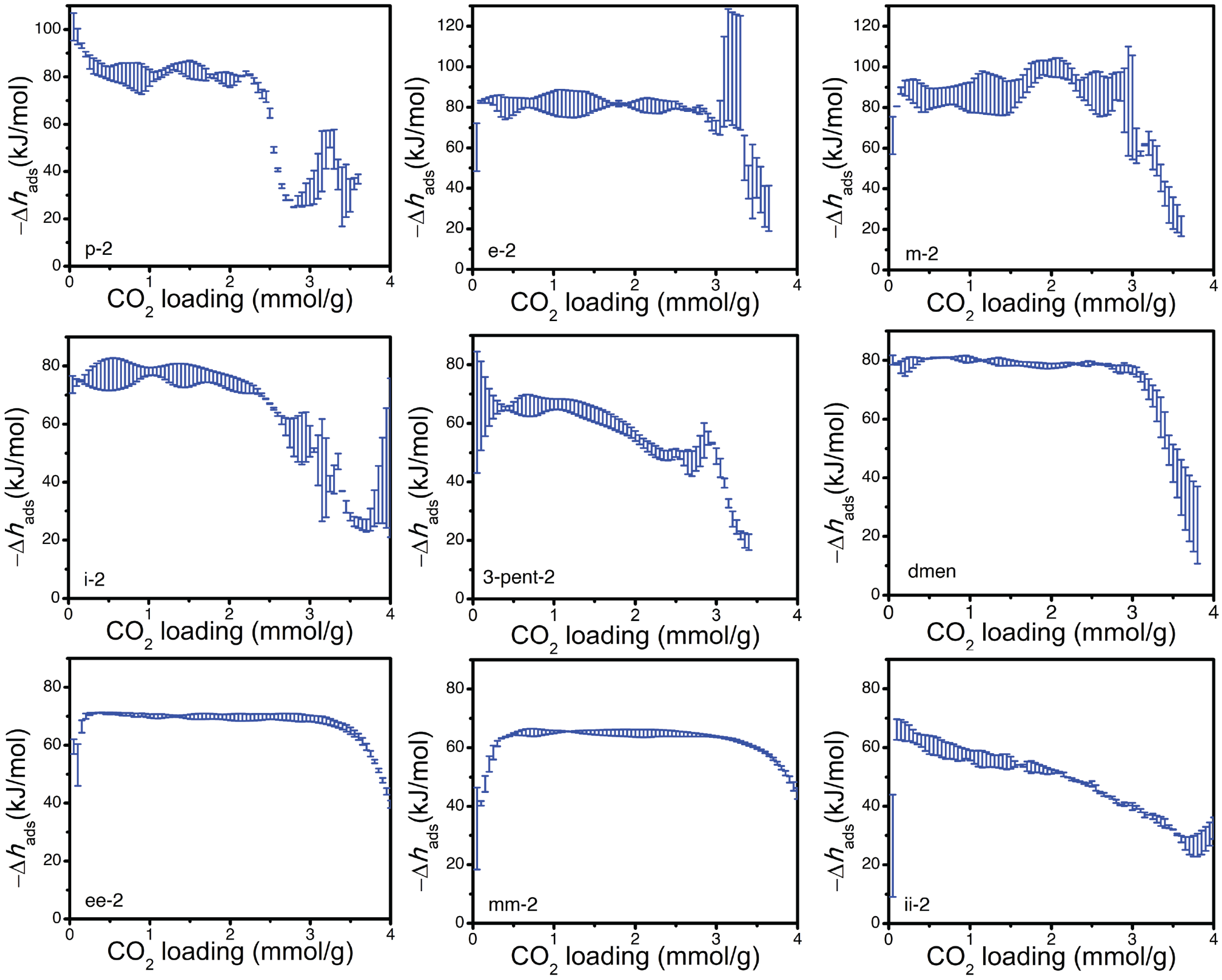


**Figure S13.** Differential heats of $CO_2$ adsorption in diamine–$Mg_2$(olz), determined using the Clausius–Clapeyron equation.

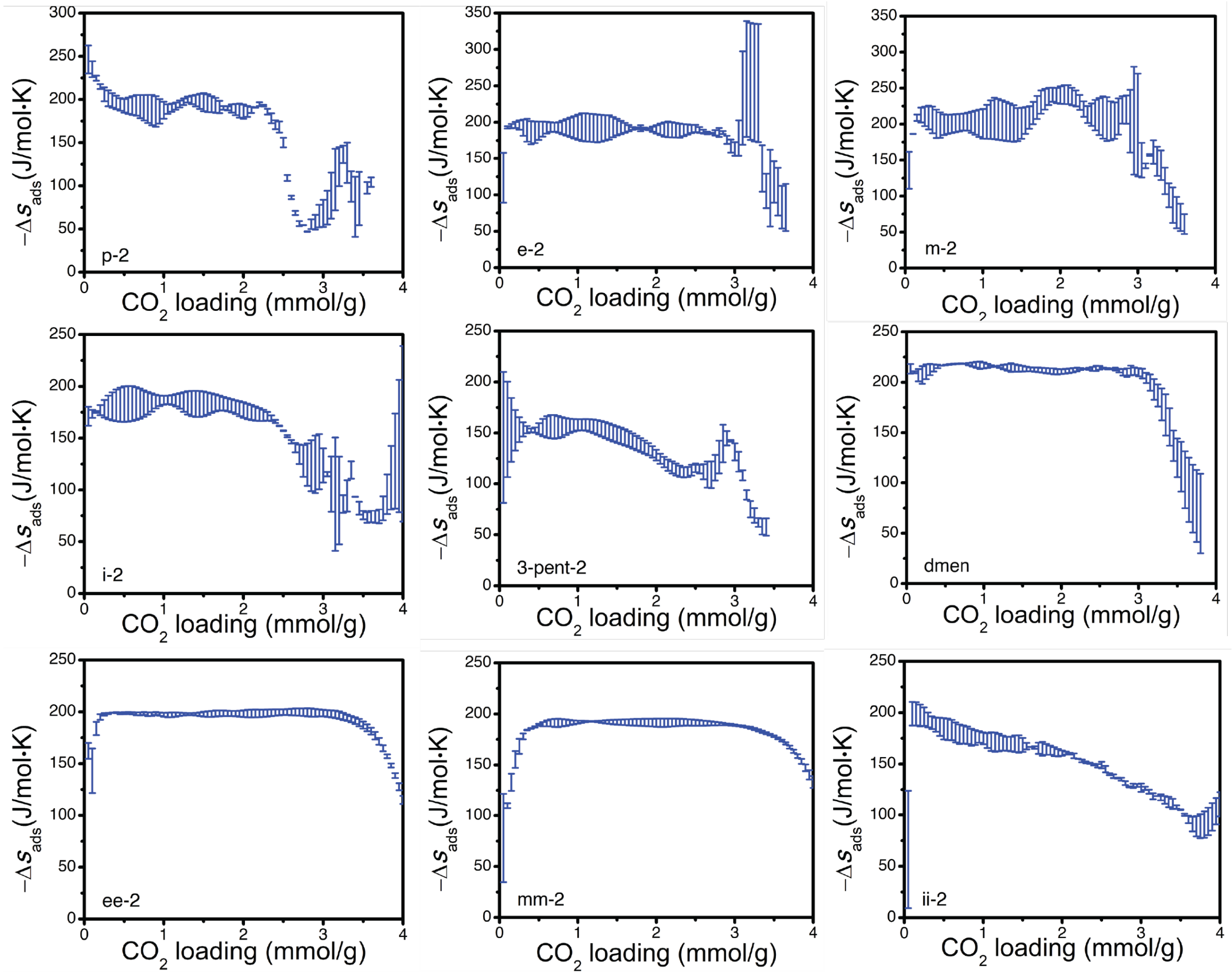


**Figure S14.** Differential entropies of $CO_2$ adsorption in diamine–$Mg_2$(olz), determined using the Clausius–Clapeyron equation.

**Table S5.** Differential enthalpies and entropies of $CO_2$ adsorption in several diamine-appended variants of $Mg_2(olz)$ at a loading of 1 mmol/g.

| ***Diamine Name*** | **$-\Delta h_{ads}$ (kJ/mol)** | **$-\Delta s_{ads}$ (J/mol·K)** |
|---|---|---|
| m-2 | 85 ± 7 | 200 ± 21 |
| e-2 | 82 ± 6 | 193 ± 17 |
| p-2 | 93 ± 11 | 222 ± 30 |
| i-2 | 77 ± 4 | 183 ± 12 |
| 3-pent-2 | 63 ± 3 | 148 ± 8 |
| dmen | 79.2 ± 0.9 | 214 ± 3 |
| ee-2 | 69.9 ± 0.8 | 198 ± 2 |
| mm-2 | 65.2 ± 0.8 | 192 ± 2 |
| ii-2 | 55 ± 2 | 169 ± 7 |

## Infrared Spectra of Diamine-Appended Metal–Organic Frameworks

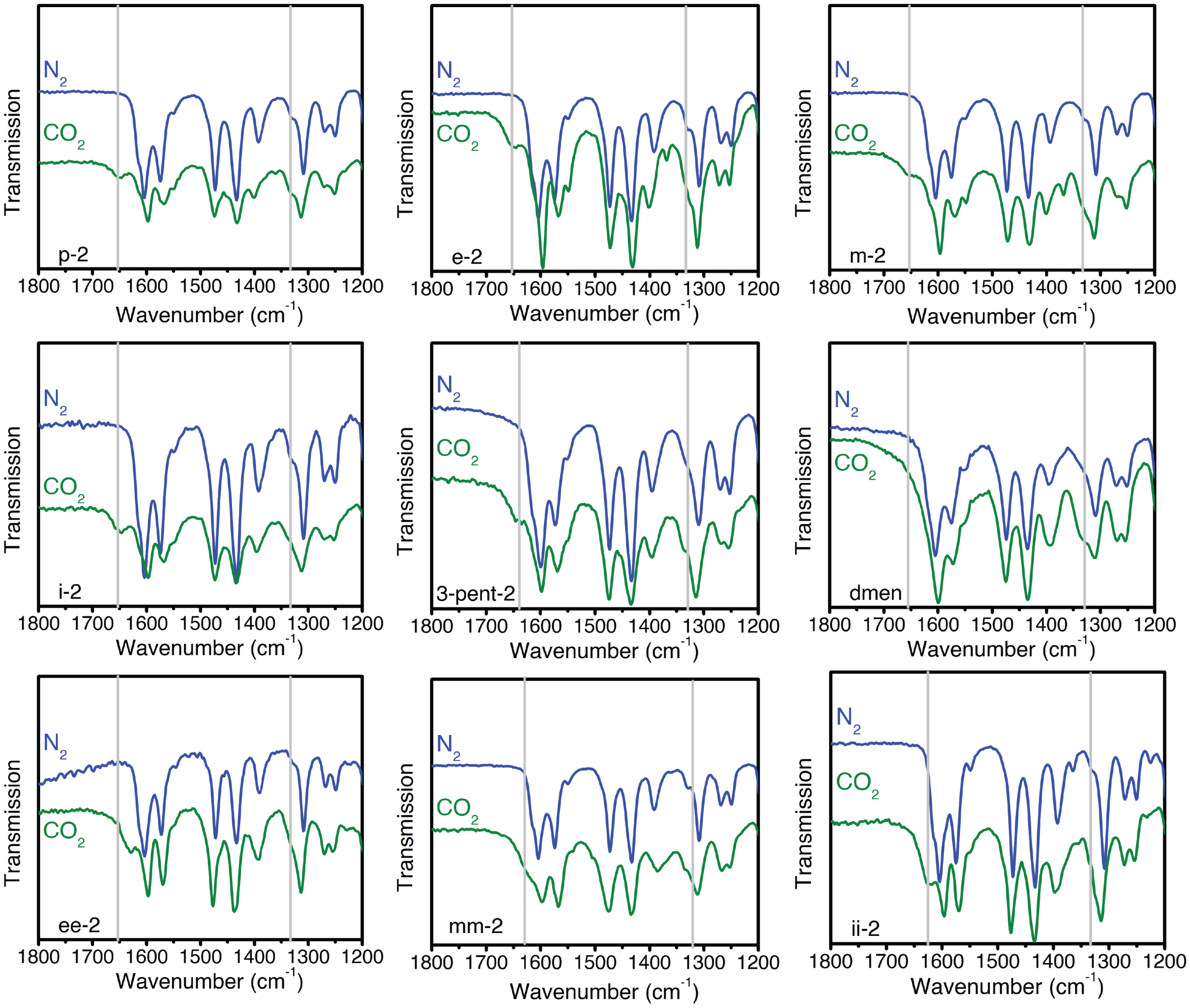


**Figure S15.** *In situ* infrared spectra at room temperature under $N_2$ (blue) and $CO_2$ (green) for activated samples of diamine–$Mg_2$(olz) discussed in this work. Gray lines mark the diagnostic carbamate ν(C–O) (1630–1690 $cm^{-1}$) and ν(C–N) (~1320 $cm^{-1}$) bands.

## 3. Characterization of ee-2–$Mg_2$(olz)

### $O_2$ and $N_2$ Adsorption Isobars and Isotherms

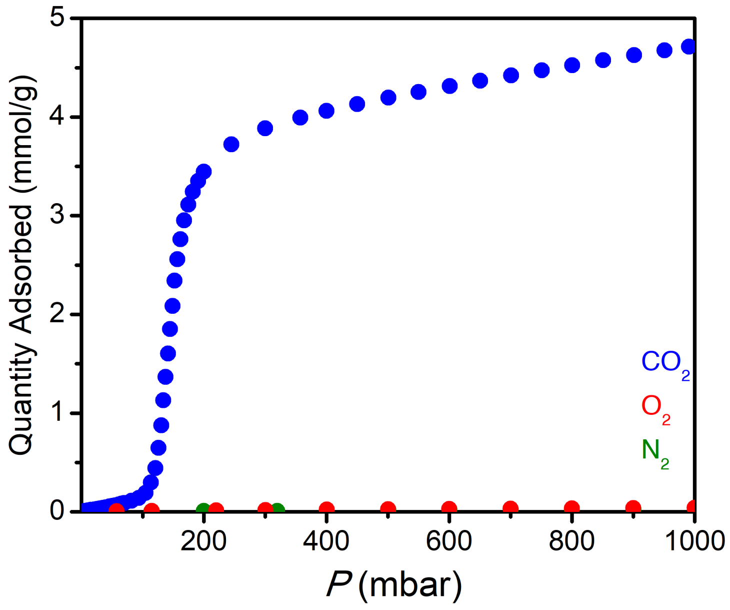


**Figure S16.** Adsorption isotherms ($CO_2$, $O_2$, and $N_2$) for ee-2–$Mg_2$(olz) collected at 40 °C.

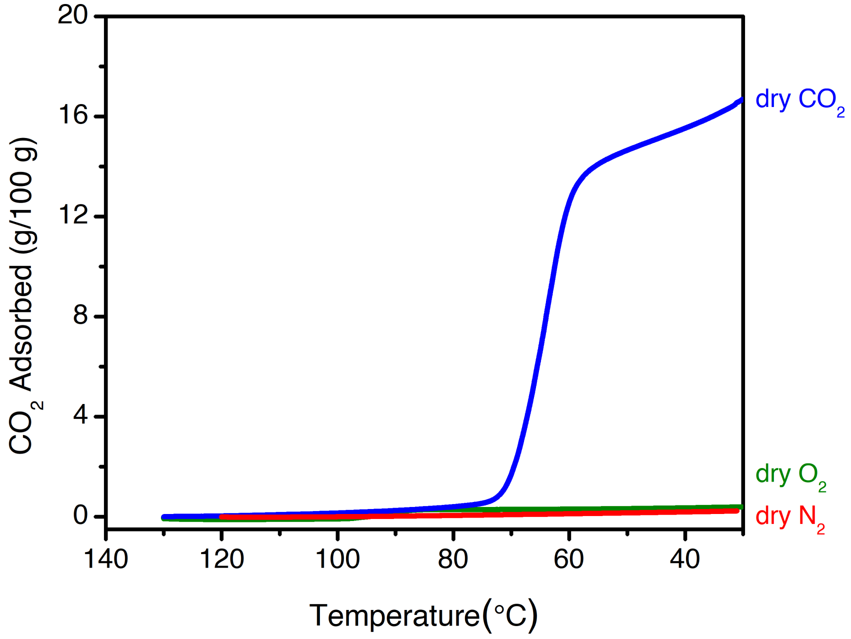


**Figure S17.** Dry $CO_2$, $O_2$, and $N_2$ adsorption isobars collected for ee-2–$Mg_2$(olz) at atmospheric pressure. A ramp rate of 1 °C/min was used.

## $CO_2$ Adsorption Isobars in Different Concentrations

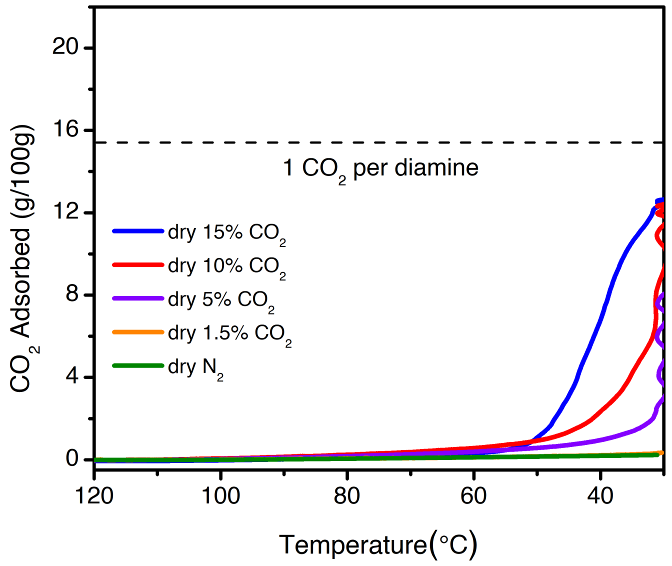


**Figure S18.** Dry 15% $CO_2$ in $N_2$ (blue), dry 10% $CO_2$ in $N_2$ (red), dry 5% $CO_2$ in $N_2$ (violet), dry 1.5 % $CO_2$ in $N_2$ (orange), and dry $N_2$ (green) adsorption isobars collected for ee-2–$Mg_2$(olz) at atmospheric pressure. A cooling ramp rate of 1 °C/min was used and once the sample reached hold at 30 °C, it was held for 1 hour at that temperature to equilibrate. The undulations in the data at 30 °C arise due to the fact that it is difficult to maintain a temperature of 30 °C in the TGA instrument.

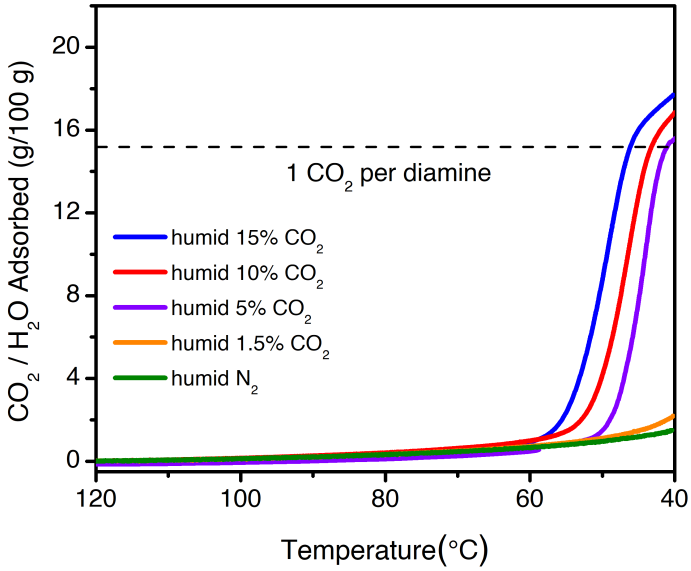


**Figure S19.** Humid (~1.5% $H_2O$) 15% $CO_2$ in $N_2$ (blue), humid 10% $CO_2$ in $N_2$ (red), humid 5% $CO_2$ in $N_2$ (violet), humid 1.5 % $CO_2$ in $N_2$ (orange), and humid $N_2$ (green) isobars of ee-2–$Mg_2$(olz) at atmospheric pressure. A ramp rate of 1 °C/min was used.

**Calculation of the Approximate Regeneration Energy of ee-2–Mg$_2$(olz)**

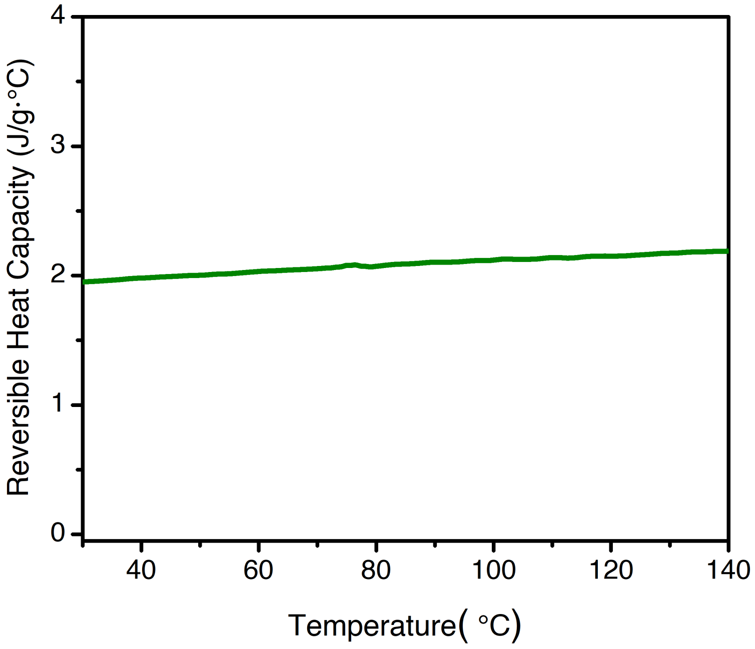


**Figure S20.** Reversible heat capacity of ee-2–Mg$_2$(olz), determined by modulated differential scanning calorimetry (DSC) under an atmosphere of He. The ramp rate was 2 °C/min. A modulation frequency of 0.75 °C/80 s was used.

Regeneration energies were calculated using the following approach:

$$\frac{MJ}{kg\ CO_2} = \frac{1}{q_{wc}} * \frac{22.72\ mol\ CO_2}{kg\ CO_2} * \frac{1\ MJ}{1000\ kJ}\left(C_p\Delta T + |\Delta h_{ads}| q_{wc}\right)$$

$C_p$ is the heat capacity in J/g$_{MOF}$·°C = kJ/kg$_{MOF}$·°C; $C_p$ of ee-2–Mg$_2$(olz) is 2.02 kJ/kg·°C.

$\Delta T$ is the temperature swing; $\Delta T$ of ee-2–Mg$_2$(olz) is 45 °C.

$\Delta h_{ads}$ is the differential enthalpy of adsorption in kJ/mol$_{CO2}$; $\Delta h_{ads}$ of ee-2–Mg$_2$(olz) is 69.9 kJ/mol.

$q_{wc}$ is the working capacity in mol$_{CO2}$/kg$_{MOF}$; $q_{wc}$ of ee-2–Mg$_2$(olz) is 3.6 mol$_{CO2}$/kg$_{MOF}$

The $C_p\Delta T$ term accounts for the sensible heat, while the $|\Delta h_{ads}|q_{wc}$ term accounts for the regeneration heat.

**Adsorption/Desorption Cycling of ee-2–$Mg_2$(olz)**

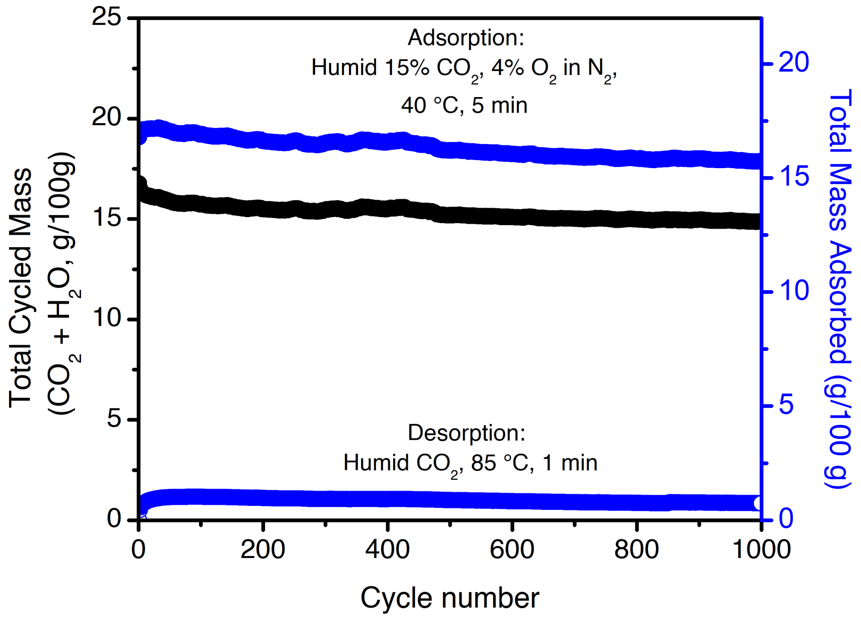


**Figure S21.** Complete data obtained over the course of 1000 humid adsorption/desorption cycles performed with ee-2–$Mg_2$(olz). Adsorption: humid 15% $CO_2$ in $N_2$, 40 °C, 5 min. Desorption: humid pure $CO_2$, 85 °C, 1 min. The baseline value of 0 g/100 g is defined as the mass after activation under humid 15% $CO_2$ in $N_2$ for 30 min at 130 °C prior to the first cycle.

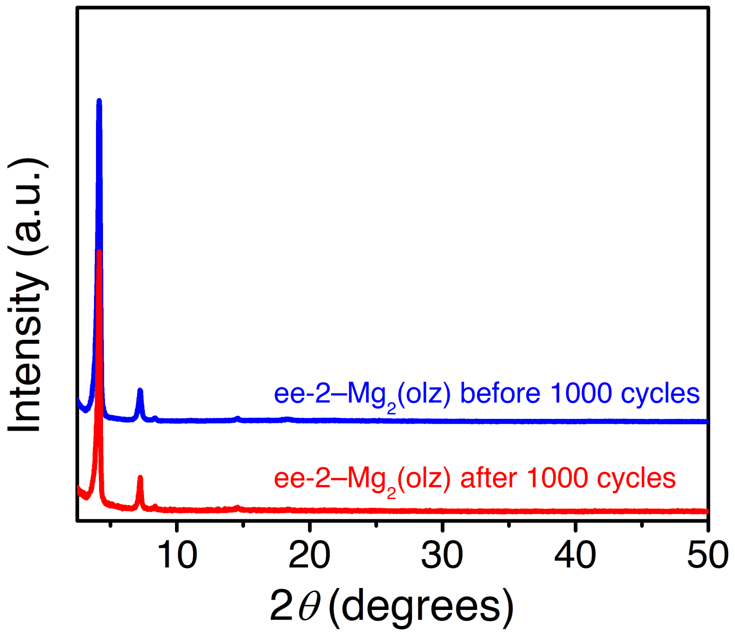


**Figure S22.** Powder x-ray diffraction patterns collected for ee-2–$Mg_2$(olz) before (blue trace) and after (red trace) 1000 cycles (CuK$\alpha$ radiation, $\lambda$ = 1.5418 Å).

**Breakthrough Experiment Details**

As-synthesized ee-2–$Mg_2$(olz) powder was filtered from the amine/toluene solution (see the Experimental Section) and then compressed into a tablet using mechanical press. The tablet was then broken into pellets using 45 mesh sieves and collected via 25 mesh sieves so that the pellet sizes were between 350 and 700 μm in diameter. These pellets were then activated on a Schlenk line with heating at 130 °C for 1 h under a continuous $N_2$ purge. The breakthrough column, comprising a 6" section of 1/4" stainless steel pipe (i.d. 0.18") with Swagelok fittings on each side, was packed with 0.58 g of activated ee-2–$Mg_2$(olz) pellets, and glass wool was added to each end of the column to secure the pellets.

The $CO_2$ capture performance of this material under multicomponent conditions was then measured via a custom-built breakthrough apparatus, containing a Parker-Porter mass flow controller, copper and stainless steel tubing (1/8") with Swagelok fittings and valves to control the gas flow, and an Agilent ADM200 Universal Flow Meter measuring the outlet gas flow rate. The outlet gas concentration was measured at 1 min intervals via an SRI Instruments 8610C gas chromatograph equipped with a Hayesep D column and TCD detector. The instrument was calibrated using a series of premixed standard tanks of varying concentrations of $CO_2$ (5, 10, 15, 20, 30, and 50%) in $N_2$ purchased from Praxair, as well as pure $CO_2$ and $N_2$. Peak integration and analysis were calculated using PeakSimple software.

The column was first activated at 130 °C under 10 sccm of He flow for 1 h, then cooled to 40 °C for measurement. The breakthrough measurements were then carried out using a 15% $CO_2$ in $N_2$ premixed cylinder from Praxair at a flow rate of 10 sccm and atmospheric pressure. The material was reactivated at 85 °C for 1 h under 10 sccm of He flow between each measurement. The $CO_2$ and $N_2$ uptake capacities ($q_i$, in mmol/g) were calculated via the following formula:

$$q_{ads} = \frac{F_{i,0}}{m\left(22.414\, {}^{cc_{STP}}\!/_{mmol}\right)} \int_0^t (1 - \frac{F_i}{F_{i,0}}) dt - \frac{\varepsilon V y_i P}{RTm},$$

where $F_{i,0}$ and $F_i$ are the inlet and outlet mass flowrates of species $i$ in sccm, $m$ is the sample mass in g, $t$ is the time since the start of the breakthrough measurement in minutes, $y_i$ is the mole fraction in the inlet gas stream, $V$ is the volume of the adsorbent column in standard cc, $P$ and $T$ are the pressure and temperature of the system, and $R$ is the universal gas constant. The void fraction of the column, $\varepsilon$, is made up of the empty space between particles due to their packing and is equal to approximately 0.4 for spherical particles of this size.[5] The error in reported capacity was calculated by propagating the standard deviation of the measured outlet flowrate, the most variable measured value, through the capacity calculation.

For humid breakthrough experiments, a fritted water bubbler was added upstream of the adsorbent column, providing an $H_2O$ concentration of ~2% assuming saturation at room temperature (20 °C). The breakthrough column was pre-saturated with $H_2O$ using 10 sccm of humid He overnight prior

to each experiment. Pre-saturation of the material was confirmed by a column of indicating Drierite downstream of the adsorption column, which was removed after saturation and before beginning the breakthrough measurement. Furthermore, the bubbler was also pre-saturated with $CO_2$ by closing off the adsorbent column and flowing the 15% $CO_2$ in $N_2$ stream through the bubbler and then through the bypass directly to the GC until a stable concentration of 15% $CO_2$ was recorded, indicating that $CO_2$ was no longer dissolving into the bubbler water, a process which typically took 30-60 min.

**Additional Solid-state Magic Angle Spinning $^{13}C$ NMR Spectra and Details**

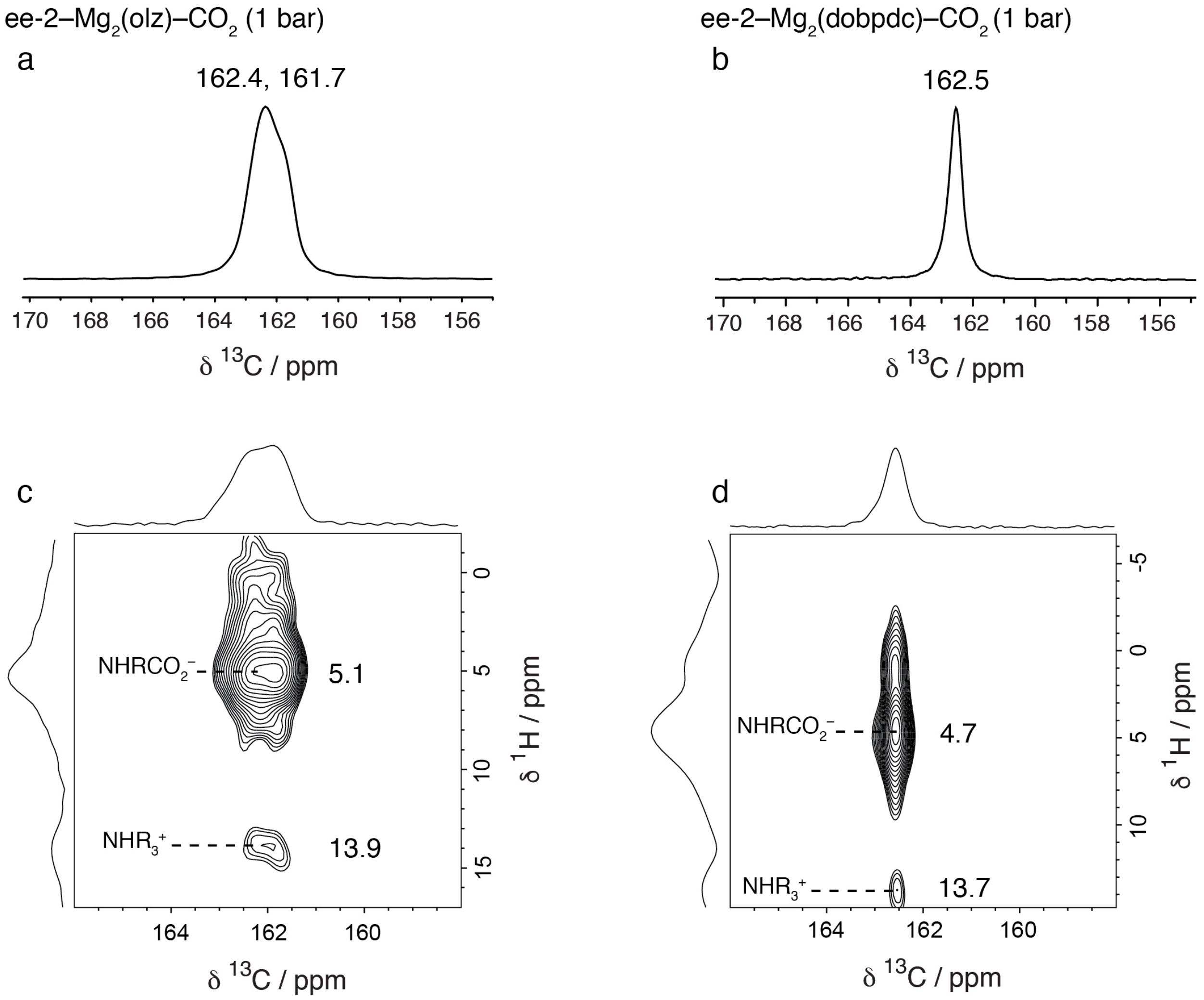


**Figure S23.** Solid-state MAS $^{1}H \rightarrow {}^{13}C$ cross-polarization spectra (16.4 T) of (a) ee-2–$Mg_2$(dobpdc) and (b) ee-2–$Mg_2$(dobpdc) dosed with 997 mbar of $CO_2$. Spectra were collected with continuous-wave decoupling of $^{1}H$. The $^{1}H \rightarrow {}^{13}C$ HETCOR (contact time 100 μs) spectra (and correlation assignments) for each sample are shown in (c) and (d), respectively.

## Van der Waals-Corrected DFT Calculations

To elucidate the effect of framework size on $CO_2$ adsorption, we performed first-principles density functional theory (DFT) calculations using a plane-wave basis and projector augmented-wave (PAW)[6-7] pseudopotentials with the Vienna ab-initio Simulation Package (VASP) code.[8-11] To include the effect of the van der Waals (vdW) dispersive interactions on binding energies, we performed structural relaxations with vdW dispersion-corrected functionals (vdW-DF2)[12] as implemented in VASP. For all calculations, we used (i) a Γ-point sampling of the Brillouin zone and (ii) a 600-eV plane-wave cutoff energy. We explicitly treated two valence electrons for Mg ($3s^2$), six for O ($2s^2 2p^4$), five for N ($2s^2 2p^3$), four for C ($2s^2 2p^2$), and one for H($1s^1$). All structural relaxations were performed with a Gaussian smearing of 0.05 eV.[13] The ions were relaxed until the Hellmann-Feynman forces are less than 0.02 eVÅ$^{-1}$.

To compute $CO_2$ binding energies, we optimized e-2–$Mg_2$(olz), ee-2–$Mg_2$(olz), e-2–$Mg_2$(dobpdc), and ee-2–$Mg_2$(dobpdc) MOFs prior to $CO_2$ adsorption ($E_{MOF}$), interacting with $CO_2$ in the gas phase ($E_{CO2}$) within a 20 Å × 20 Å × 20 Å cubic supercell, and all MOFs with one equivalent of adsorbed $CO_2$ per diamine ($E_{CO_2\text{–}MOF}$) using vdW-corrected DFT. The binding energies ($E_B$) are obtained via the difference:

$$E_{\mathrm{B}} = E_{\mathrm{CO_2-MOF}} - \left(E_{\mathrm{MOF}} + E_{\mathrm{CO_2}}\right)$$

For NMR simulations, we performed structural relaxations with (i) a 1000 eV plane-wave cutoff energy, (ii) a 0.01 eVÅ$^{-1}$ force criterion, (iii) a 1 × 1 × 3 k-point, and (iv) a $10^{-7}$ eV self-consistency criterion. In these input criteria, the isotropic chemical shielding ($\sigma_{iso}$) is converged within 0.1 ppm. To compare our computed values to the experimental chemical shifts ($\delta_{iso}$), we need the $\sigma_{ref}$ values for $^1$H, $^{13}$C, and $^{15}$N since the isotropic chemical shift ($\delta_{iso}$) is obtained from $\delta_{iso} = -(\sigma_{iso} - \sigma_{ref})$ where $\sigma_{ref}$ is a reference value. To do this, we used the previously-determined values ($^1$H($\sigma_{ref}$) = 31.4 ppm, $^{13}$C($\sigma_{ref}$) = 160.1 ppm, $^{15}$N($\sigma_{ref}$) = 21.9 ppm). [14]

**Table S6. Computed lattice parameters and $CO_2$ binding energies of e-2–$Mg_2$(olz), ee-2–$Mg_2$(olz), e-2–$Mg_2$(dobpdc), and ee-2–$Mg_2$(dobpdc).**

| | *a* (Å) | *c* (Å) | $E_B$ (kJ/mol) |
|---|---|---|---|
| e-2–$Mg_2$(olz) | 25.541 | 6.982 | −88.6 |
| $CO_2$–e-2–$Mg_2$(olz) | 25.547 | 6.921 | |
| e-2–$Mg_2$(dobpdc) | 21.869 | 7.122 | −81.8 |
| $CO_2$–e-2–$Mg_2$(dobpdc) | 19.934 | 6.979 | |
| ee-2–$Mg_2$(olz) | 25.550 | 6.849 | −66.4 |
| $CO_2$–ee-2–$Mg_2$(olz) | 25.138 | 7.106 | |
| ee-2–$Mg_2$(dobpdc) | 21.919 | 6.965 | −49.3 |
| $CO_2$–ee-2–$Mg_2$(dobpdc) | 21.775 | 7.087 | |

**Table S7. Calculated and Experimental NMR chemical shifts for e-2–$Mg_2$(olz), ee-2–$Mg_2$(olz), e-2–$Mg_2$(dobpdc), and ee-2–$Mg_2$(dobpdc).**

| | e-2–$Mg_2$(olz) | e-2–$Mg_2$(dobpdc) | ee-2–$Mg_2$(olz) | ee-2–$Mg_2$(dobpdc) |
|---|---|---|---|---|
| | Calc | Calc | Calc (Exp) | Calc (Exp) |
| δ $^{13}C$ (ppm) | | | | |
| $COO^{-}$ | 166.2 | 164.0 | 164.8 (162.4) | 164.9 (162.5) |
| δ $^{1}H$ (ppm) | | | | |
| $NHCOO^{-}$ | 3.8 | 5.6 | 3.8 (5.1) | 5.4 (4.7) |
| $NH_2R_2^{+}$ | 12.9, 8.6 | 7.7, 7.8 | - | - |
| $NHR_3^{+}$ | - | - | 13.4 (13.9) | 13.0 (13.7) |
| δ $^{15}N$ (ppm) | | | | |
| $RNHCOO^{-}$ | 83.9 | 83.8 | 93.5 | 92.1 |
| $R_2NH_2\cdots COO^{-}$ | 46.3 | 45.5 | - | - |
| $R_3NH\cdots COO^{-}$ | - | - | 60.1 | 68.4 |

## Crystallographic Data

**Table S8. Crystallographic data for diamine–$Mg_2$(olz), determined from Pawley refinement of synchrotron powder x-ray diffraction data.**

| | Space group | a / Å | c / Å | V / Å$^3$ | $R_{wp}$ (%) | $R_{exp}$(%) |
|---|---|---|---|---|---|---|
| p-2–$Mg_2$(olz) | $P3_221$ | 24.458(2) | 6.531(3) | 3383(1) | 3.16 | 1.84 |
| e-2–$Mg_2$(olz) | $P3_221$ | 24.468(2) | 6.525(3) | 3393(2) | 2.96 | 1.57 |
| m-2–$Mg_2$(olz) | $P3_221$ | 24.432(2) | 6.543(2) | 3382(1) | 3.39 | 1.64 |
| i-2–$Mg_2$(olz) | $P3_221$ | 24.451(1) | 6.538(3) | 3385(2) | 2.78 | 1.66 |
| 3-pent-2–$Mg_2$(olz) | $P3_221$ | 24.34(2) | 6.59(1) | 3380(8) | 4.10 | 1.44 |
| dmen–$Mg_2$(olz) | $P3_221$ | 24.255(8) | 6.484(3) | 3304(3) | 3.91 | 2.12 |
| ee-2–$Mg_2$(olz) | $P3_221$ | 24.352(2) | 6.528(2) | 3352(1) | 2.03 | 2.46 |
| mm-2–$Mg_2$(olz) | $P3_221$ | 24.413(2) | 6.523(2) | 3366(1) | 3.15 | 1.77 |
| ii-2–$Mg_2$(olz) | $P3_221$ | 24.271(3) | 6.504(2) | 3318(1) | 2.47 | 1.95 |

**Table S9. Crystallographic Data for $CO_2$–diamine–$Mg_2$(olz), determined from Pawley refinement of synchrotron powder x-ray diffraction data.**

| | Space group | a / Å | c / Å | V / Å$^3$ | $R_{wp}$ (%) | $R_{exp}$(%) |
|---|---|---|---|---|---|---|
| $CO_2$–p-2–$Mg_2$(olz) | $P3_221$ | 24.564(3) | 6.558(4) | 3426(2) | 2.74 | 1.65 |
| $CO_2$–e-2–$Mg_2$(olz) | $P3_221$ | 24.355(3) | 6.532(5) | 3355(3) | 2.53 | 1.50 |
| $CO_2$–m-2–$Mg_2$(olz) | $P3_221$ | 24.335(2) | 6.508(4) | 3338(2) | 2.85 | 1.54 |
| $CO_2$–i-2–$Mg_2$(olz) | $P3_221$ | 24.404(2) | 6.558(3) | 3383(1) | 3.04 | 2.70 |
| $CO_2$–3-pent-2–$Mg_2$(olz) | $P3_221$ | 24.50(1) | 6.632(8) | 3447(5) | 3.42 | 1.36 |
| $CO_2$–dmen–$Mg_2$(olz) | $P3_221$ | 24.079(4) | 6.665(5) | 3347(2) | 3.80 | 2.02 |
| $CO_2$–ee-2–$Mg_2$(olz) | $P3_221$ | 24.255(2) | 6.495(4) | 3309(2) | 1.86 | 1.88 |
| $CO_2$–mm-2–$Mg_2$(olz) | $P3_221$ | 24.216(2) | 6.452(4) | 3276(2) | 2.95 | 1.67 |
| $CO_2$–ii-2–$Mg_2$(olz) | $P3_221$ | 24.350(3) | 6.559(3) | 3367(2) | 2.09 | 1.88 |

## References


1. Hall, H. K., Jr., Correlation of the Base Strengths of Amines. *J. Am. Chem. Soc.* **1957**, *79* (20), 5441-5444.
2. SciFinder; Chemical Abstracts Service: Columbus, OH; p*Ka*; RN 624-78-2, 109-89-7, 20193-20-8, 19961-27-4, 39190-77-7, 75-64-9, 598-56-1, 121-44-8, 7087-68-5; https://scifinder.cas.org (accessed Dec 5, 2022); Calculated using Advanced Chemistry Development (ACD/Labs) Software V11.02 (© 1994-2022 ACD/Labs).
3. Weiss, J. N., The Hill equation revisited: uses and misuses. *The FASEB Journal* **1997**, *11* (11), 835-841.
4. MATLAB. (2022). Version R2022a. Natick, Massachusetts: The MathWorks Inc.
5. Mueller, G. E., Radial void fraction distributions in randomly packed fixed beds of uniformly sized spheres in cylindrical containers. *Powder Technol.* **1992**, *72* (3), 269-275.
6. Blöchl, P. E., Projector augmented-wave method. *Phys. Rev. B* **1994**, *50* (24), 17953-17979.
7. Kresse, G.; Joubert, D., From ultrasoft pseudopotentials to the projector augmented-wave method. *Phys. Rev. B* **1999**, *59* (3), 1758-1775.
8. Kresse, G.; Furthmüller, J., Efficient iterative schemes for ab initio total-energy calculations using a plane-wave basis set. *Phys. Rev. B* **1996**, *54* (16), 11169-11186.
9. Kresse, G.; Hafner, J., Ab initio molecular dynamics for liquid metals. *Phys. Rev. B* **1993**, *47* (1), 558-561.
10. Kresse, G.; Hafner, J., Ab initio molecular-dynamics simulation of the liquid-metal--amorphous-semiconductor transition in germanium. *Phys. Rev. B* **1994**, *49* (20), 14251-14269.
11. Kresse, G.; Furthmüller, J., Efficiency of ab-initio total energy calculations for metals and semiconductors using a plane-wave basis set. *Comput. Mater. Sci.* **1996**, *6* (1), 15-50.
12. Lee, K.; Murray, É. D.; Kong, L.; Lundqvist, B. I.; Langreth, D. C., Higher-accuracy van der Waals density functional. *Phys. Rev. B* **2010**, *82* (8), 081101.
13. Elsässer, C.; Fähnle, M.; Chan, C. T.; Ho, K. M., Density-functional energies and forces with Gaussian-broadened fractional occupations. *Phys. Rev. B* **1994**, *49* (19), 13975-13978.
14. Siegelman, R. L.; Milner, P. J.; Forse, A. C.; Lee, J.-H.; Colwell, K. A.; Neaton, J. B.; Reimer, J. A.; Weston, S. C.; Long, J. R., Water Enables Efficient $CO_2$ Capture from Natural Gas Flue Emissions in an Oxidation-Resistant Diamine-Appended Metal–Organic Framework. *J. Am. Chem. Soc.* **2019**, *141* (33), 13171-13186.
